# Mitochondrial remodeling and metabolic reprogramming drive long-term salinity adaptation in *Tetrahymena thermophila*

**DOI:** 10.1128/msystems.01549-25

**Published:** 2025-12-23

**Authors:** Fengyu Yuan, Wenyu Li, Aiyun Li, Ting Tang, Yuming Zhang, Song Xie, Fengchao Li, Fengsong Liu

**Affiliations:** 1 The Key Laboratory of Zoological Systematics and Application, College of Life Sciences, Hebei University 162640 , Baoding, China; 2 Hebei Basic Science Center for Biotic Interaction, Hebei University 56667, Baoding, China; 3 Engineering Research Center of Ecological Safety and Conservation in Beijing-Tianjin-Hebei (Xiong'an New Area) of MOE, Baoding, China; Ocean University of China, Qingdao, Shandong Province, China

**Keywords:** aquatic salinization, experimental evolution, osmoregulation, mitochondrial energetics, oxidative stress adaptation, lipid storage repurposing

## Abstract

Salinization of inland waters, driven by climate change and human activities, poses a major threat to aquatic ecosystems. While species can swiftly adapt to environmental stress, the molecular mechanisms underpinning this adaptation remain to be fully elucidated. This study seeks to clarify the complex adaptive strategies employed by the freshwater ciliate *Tetrahymena thermophila* in response to chronic salt stress through the methodologies of experimental evolution and multi-omics integration. The findings indicate that three lineages adapted to salt (ST-4, ST-8, and ST-12), which evolved under a regime of increasing NaCl concentration, demonstrated a trade-off between delayed growth and osmotic resilience. Transcriptomic and proteomic analyses revealed key evolutionary priorities, including (i) the co-upregulation of pathways related to DNA replication, glutathione metabolism, and endoplasmic reticulum (ER) protein processing, (ii) the suppression of lipid catabolism alongside the accumulation of lipid droplets mediated by START2, and (iii) mitochondrial remodeling through the expansion of ER contacts to sustain ATP production. Interestingly, the adaptation to salt appears to tolerate genome instability induced by replication stress through the dysregulation of replisome components, specifically the upregulation of Prim1 and downregulation of LIG, while also evading antioxidant defenses via the compartmentalization of oxidative damage. These results contribute to a framework in which protists effectively balance lipid-mediated osmoregulation, controlled mutagenesis, and organelle metabolism to navigate salinity challenges, thereby offering predictive insights into microbial adaptation thresholds within evolving ecosystems.

**IMPORTANCE:**

Salinization of inland waters is a growing concern due to climate change and human activities. Understanding how organisms adapt to saline environments is vital. *Tetrahymena thermophila*, a model organism, was studied to explore its adaptation mechanisms. The findings show that through gene regulation, it can acclimate to high salt conditions. The role of mitochondria in metabolic reprogramming during this process is significant. This research contributes to a more profound understanding of how organisms adapt to saline stress and the molecular mechanisms underlying such adaptations, which may aid in predicting and managing the impacts of salinization on aquatic ecosystems.

## INTRODUCTION

Freshwater salinization is accelerating globally, driven by human activities such as industrial wastewater discharge, agricultural runoff, and urbanization (e.g., road salts can elevate salinity concentrations in freshwater ecosystems to 200–5,000 mg Cl^−^/L) and exacerbating natural salinity fluctuations linked to extreme climate (1–3). This ionic pollution has been shown to diminish water quality and disrupt the osmotic regulation of freshwater organisms. For example, at a NaCl concentration of 860 mg/L, the reproductive lifespan of freshwater zooplankton was reduced by 23% to 83% (4–6). While some salt-tolerant taxa displace sensitive species, such as freshwater insects Ephemeroptera (mayflies), species richness declines, posing a threat to ecosystem resilience (7–9). Notably, in contrast to metazoans, the adaptive strategies of microbial eukaryotes, which are pivotal drivers of biogeochemical cycles, remain under-researched.

Ciliates, comprising more than 10,000 species (https://www.gbif.org/species/7765738), are predominant in microbial eukaryotic communities across a wide range of salinity gradients, from freshwater lakes to Tibetan Plateau sediments to salt marshes (e.g., *Fabrea salina* can withstand conditions of 35–180 ppt) (10–15). Ciliates demonstrate extreme osmotic adaptability unmatched in most eukaryotes. For instance, the halophile *Enchelyothrix muria* thrives in the saturated saline waters of Lake Mahalu in Iran, while *Strombidium sulcatum* regulates its osmotic adaptation through compatible solutes, such as glycine betaine (16–19). Recent studies on *Fabrea salina* have shown an expansion of gene families for DNA replication proteins and mitochondrial biogenesis, and the accumulation of phosphatidic acid may play an important role in resistance to high osmotic pressure (16, 20, 21). The majority of research on salt stress in salt-tolerant microorganisms has focused on prokaryotic organisms. For example, the halophilic bacteria *Pontixanthobacter* and *Allopontixanthobacter* demonstrate a notable accumulation of genes associated with osmotic regulation, membrane permeability, and signal transduction (22). The halophilic archaeon *Halorutilus salinus* primarily adapts to elevated salt concentrations through osmotic protection mechanisms (23). *Euplaesiobystra salpumilio*, a novel halophilic amoeboflagellate, exhibits distinct intracellular Na^+^ and K^+^ accumulation patterns with salinity-dependent salt-in and salt-out strategies, yet its molecular mechanisms and regulatory logic remain unclear, requiring further investigation (24). These mechanisms position ciliates as a model for extreme microbial biology, thereby linking cellular physiology with adaptability at the ecosystem level.

*Tetrahymena thermophila* is a widely used model organism in ciliates. The whole genome of this organism has been assembled and annotated with high precision, and its unique nuclear dimorphism can provide a natural system for analyzing genome dynamics and epigenetic regulation under salt stress (25–27). In terms of technical feasibility, its genetic manipulation tools are mature, which can efficiently carry out gene function verification and facilitate the analysis of osmoregulation-related mechanisms (28). In terms of environmental stress response, *T. thermophila* has a clear and observable response mechanism to ionic stress. Under heavy metal stress, this organism induces the expression of metallothioneins (MTs), which are cysteine-rich (22–54 proteins) and have a significantly higher binding capacity for metal ions than the classical MTs*,* providing a typical example of ion homeostasis strategies in eukaryotes (29). Meanwhile, as a cell wall-less eukaryote, its cell membrane can directly perceive the environmental ion changes, the stress response is more direct, and the related physiological indicators (e.g., intracellular ion concentration, oxidative stress level) are easy to be detected, which is a feature that gives it a significant advantage in the study of salt stress mechanism (30–32). In addition, as a key microorganism in freshwater ecosystems, the study of its adaptive mechanism can be directly related to the impact of salinity on the ecosystem, which is highly compatible with the background of this study (33).

In this study, a 3-year experimental evolution approach (~6,750 generations) was employed to reveal the high-salinity adaptation strategies in *T. thermophila*. This approach involved the generation of stable lineages that thrived at 4–12 g/L NaCl through sequential selection of four survival pillars: (i) membrane lipidome remodeling and transporter network optimization. (ii) Mitochondrial metabolic rewiring through organelle networking. (iii) Redox system repurposing via spatial damage compartmentalization. (iv) Replication stress-mediated genomic plasticity. By integrating multi-omics profiling with functional phenotyping, this systematic dissection reveals how synchronized alterations across membrane architecture, bioenergetic circuits, antioxidant hierarchies, and DNA replication fidelity collectively drive extremophile survival. The findings delineate protistan halotolerance as a dynamically coordinated adaptation process integrating organelle remodeling, metabolic reprogramming, and genomic plasticity, thus bridging critical gaps in microbial stress biology while identifying novel biomarkers for assessing ecosystem resilience in salinizing environments.

## MATERIALS AND METHODS

### *T. thermophila* culture

*T. thermophila* CU428 was provided by the National Aquatic Biological Resource Center, Wuhan, China. The cells were cultured in SPP medium, which consisted of 1% proteose peptone, 0.2% glucose, 0.1% yeast extract, 0.03% Fe-EDTA, 100 U/mL penicillin, 100 mg/L streptomycin sulfate, and 0.025 mg/L amphotericin B at 30°C (31).

### NaCl cytotoxicity and growth inhibition assay

*T. thermophila* cultures were exposed to NaCl gradients (0–12 g/L) in SPP medium to assess salinity tolerance. The cells were initially inoculated at a density of 5,000 cells/mL in conical flasks and then incubated for a period of 24 h under standard conditions. Cell density and viability were then quantified using an automated cell counter (DeNovix CellDrop BF) in accordance with established protocols (34). Morphometric analyses included differential interference contrast microscopy (Leica DMi8) and ImageJ-based quantification of cellular dimensions (length, width, projected area) according to methodologies previously published (35). A total of 100 randomly selected cells per treatment group were analyzed across triplicate independent experiments.

### Salt-tolerant strain development and phenotypic characterization

The salt-tolerant (ST) strains of *T. thermophila* were developed through a long-term experimental evolution approach under incremental NaCl selection pressure over a total period of 3 years.

#### Adaptation process

The wild-type (WT) strain was subjected to a stepwise acclimation process. The initial adaptation cycle involved inoculating cells at a density of 5,000 cells/mL in SPP medium supplemented with 4 g/L NaCl. The cultures were maintained with routine medium renewal every 2 days (48-h intervals), during which cells were re-inoculated at the same initial density into fresh selective medium. The NaCl concentration was systematically increased at a rate of 1 g/L per month. Upon reaching the first target concentration of 8 g/L, the population was maintained under this selective pressure for a consolidation period of 6 months to ensure stable phenotypic fixation. The salinity was then progressively increased again at the same rate until the final target of 12 g/L was achieved, followed by a second consolidation period of 6 months. This rigorous, multi-year selection regimen resulted in the establishment of three distinct, clonally isolated salt-tolerant lineages, designated as ST-4, ST-8, and ST-12, corresponding to their respective final adaptation NaCl concentrations (36).

#### Phenotypic characterization

Growth kinetics of the WT and ST lineages were assessed in their respective NaCl environments via non-invasive optical density monitoring at OD₄₀₀, measured at 4-h intervals using a Synergy HTX Multimode Reader (BioTek, USA). Cellular morphology was quantified by imaging paraformaldehyde-fixed samples under phase-contrast microscopy, with subsequent morphometric analysis (cell length, width, and projected area) performed using ImageJ software (≥100 cells per group). All ST strains were maintained via subculturing every 2 days in their adaptation media, with cell density routinely verified using the DeNovix CellDrop BF automated cell counter to ensure consistency.

Growth kinetic parameters, including the maximum specific growth rate (μ) and generation time (Tg), were determined from the OD₆₀₀ data during the exponential growth phase. Briefly, linear regression was applied to the plot of the natural logarithm of OD against time for each replicate, and the slope was taken as μ. The Tg was calculated as ln(2)/μ. The detailed methodology and results are provided in the Supplementary Material (Table S1).

### Cell viability assessment

Cellular vitality was quantified using a triplex fluorescence assay. WT and ST *T. thermophila* cells from mid-log phase cultures were harvested by gentle centrifugation. For each fluorescence assay, a standardized total of 1.5 × 10⁶ cells from each biological replicate (*n* = 6) was resuspended in 1 mL of the respective dye solution. After incubation, a 100 µL aliquot of this suspension was dispensed into individual wells of a 96-well microplate for fluorescence measurement, ensuring that the signal from an identical number of treated cells was compared across conditions.

Fluorescence signals for neutral red (NR, 33 μg/mL, λex/λem = 530/645 nm)*,* propidium iodide (PI, 10 μg/mL, λex/λem = 535/620 nm), and AlamarBlue (AB, λex/λem = 560/590 nm) were measured using a SpectraMax i3x multi-mode microplate reader (Molecular Devices, USA). The use of this dedicated fluorescence plate reader, coupled with the standardized cell input, guaranteed consistent and highly sensitive quantification of the distinct physiological metrics provided by each dye (37).

### RNA sequencing and transcriptome analysis

Total RNA from WT and ST strains (ST-8/ST-12) was sequenced on Illumina NovaSeq 6000 (Novogene Bioinformatics Technology Co. Ltd.) with 150-bp paired-end reads (Q30 > 90%, 40 M/sample). Reads were aligned to the *T. thermophila* genome (assembly 2024, GCA_037492255.1) (25) via BWA-MEM (v0.7.5a, -M -t 8), quantified by FPKM. DESeq2 (v1.38.3) identified differentially expressed genes (DEGs) (|log2FC| ≥ 1.0, adj. *P* ≤ 0.05). Enriched pathways (clusterProfiler v4.8.1) were visualized in Cytoscape (v3.9.1).

To validate the transcriptomic data obtained from RNA sequencing, we conducted quantitative reverse-transcription PCR (qRT-PCR) analysis using cDNA templates synthesized from total RNA of both WT and salt-tolerant strains (ST-8 and ST-12). Gene-specific primers were designed for nine selected genes, including ABC2 (TTHERM_00137850, ABC transporter), MSF (TTHERM_00456910, MFS transporter), TP (TTHERM_00723490, transmembrane protein), ACAD (TTHERM_00188450, acyl-CoA dehydrogenase), ACOX (TTHERM_00586710*,* peroxisomal acyl-CoA oxidase), GSTm46 (TTHERM_00689990), GSTm34 (TTHERM_00661650)*,* PCNA (TTHERM_01107420*,* proliferating cell nuclear antigen), and LIG4 (TTHERM_00387050, DNA ligase IV). The qRT-PCR was performed following an established method with three biological replicates per strain (38). The 17S rRNA was used as an internal control for data normalization (39), and relative expression levels were calculated using the 2^–ΔΔCt^ method (40) with normalization to the WT control group. All primer sequences used in this study are listed in Table S1.

### Quantitative proteomic profiling

Quantitative proteomic analysis was performed by SHANGHAI BIOTREE BIOTECH CO., LTD (Shanghai, China) using data-independent acquisition (DIA) on a timsTOF Pro2 mass spectrometer. Proteins of WT and ST-8 (200 μg/sample) were digested with Trypsin Gold (Promega, 1:30, 16 h), separated on a C18 column (75 μm × 15 cm, 1.9 μm) via 44 min ACN/0.1% FA gradient (5%–35% B). DIA-PASEF data (timsTOF Pro2, m/z 350–1,250, 25 MS/MS cycles) were analyzed in Spectronaut (v17.1) against the UniProt database (2024-05 release). Quantification used MaxLFQ normalization (the *P* value was found to be less than 0.05, and the fold change was either less than 0.83 or greater than 1.2).

### START2 knockdown and lipid droplet quantification

RNA interference targeting the START2 was performed via bacterial feeding following established protocols (41). Log-phase HT115 bacteria were used for co-culture, which expressed dsRNA targeting START2, the exogenous GFP (*Escherichia coli* expressing dsRNA of green fluorescent protein), the endogenous KIN gene (TTHERM_00481220, serving as a non-targeting control), or containing the empty L4440 vector (to control for any effects of the vector or feeding procedure). All primer sequences used for vector construction and qRT-PCR validation are listed in Table S1.

For lipid droplet analysis, RNAi-treated cells were fixed in 1% paraformaldehyde (10 min), stained with BODIPY 493/503 (5 μM, 20 min, RT) and DAPI (1 μg/mL, 10 min), then imaged via confocal microscopy (λ_ex_/λ_em_ = 488/510 nm). Lipid droplet number and size distribution were quantified from ≥100 cells per group using ImageJ (v1.53). The number of droplets (≥100 cells/group, replicated three times [42]) was analyzed using ImageJ (Fiji) (42).

### Comet assay for DNA damage quantification

DNA damage was quantified via alkaline comet assay (43). *T. thermophila* cells were embedded in 0.75% low-melting agarose and cast onto microscope slides. Slides underwent sequential lysis (4°C, 1 h), alkaline unwinding (pH > 13, 30 min), and electrophoresis (25 V, 15 min at 4°C) before neutralization (15 min). DNA was stained with propidium iodide (20 μg/mL) and visualized using confocal microscopy (Leica TCS SP8). Olive tail moment (OTM) was used to assess DNA damage (*n* ≥ 100) using the OpenComet plugin in ImageJ, and the experiment was repeated three times independently.

### Mitochondrial membrane potential assessment

Mitochondrial membrane potential (MMP) was assessed using the JC-1 probe (Beyotime C2006). Cells were incubated with JC-1 (10 μg/mL, 40 min, 12°C, dark), washed thrice with PBS (4°C, 500 × *g*, 5 min), and analyzed on HITACHI F-4600 (λex/em: 514/529 nm [monomer], 585/590 nm [aggregate]). MMP = aggregate/monomer ratio (six replicates).

### ATP quantification and energy charge analysis

A total of 2.5 × 10⁶ cells of WT, ST-4, ST-8, and ST-12 were collected and ultrasonically disrupted by adding 0.4 M HClO₄, and then centrifuged at 12,000 × *g* for 10 min. The supernatant was neutralized by adding K₂CO₃/methanol solution (0.8 M:20%). Following centrifugation at 12,000 × *g*, the resultant material was collected and filtered through a 0.22 μm membrane filter. Analysis was then undertaken by HPLC (C18 column, mobile phase consisted of 0.18 M KH_2_PO_4_ and 5% methanol, with a flow rate of 0.8 mL/min and λ = 254 nm). ATP concentration was quantified via external calibration curves. Energy charge was calculated as (ATP + 0.5 ADP)/(ATP + ADP + AMP) (44), with triplicate biological replicates analyzed.

### Assessment of acute mitochondrial stress and extreme salt tolerance

To characterize the acute stress response in WT cells, they were exposed to sub-lethal concentrations of NaCl (4 or 8 g/L) for 24 h. Following exposure, mitochondrial respiratory function (complex I, II, IV activities, and respiratory control ratio—RCR) was assessed using O2K (detailed methods are described in Method 1.3 of the Supplementary Material). Mitochondrial morphology was quantified from transmission electron microscopy (TEM) images of at least 95 cells per group using ImageJ software.

To validate long-term adaptation and assess extreme tolerance, both WT and salt-adapted (ST) strains were challenged with lethal concentrations of NaCl (12, 16, and 18 g/L) for 48 h. Survival and growth were visually assessed and scored as +++ (robust growth), ++ (inhibited growth but surviving), + (viable but no growth), and − (non-viable).

### Statistical analysis

Data (mean ± SD) analyzed by one-way ANOVA (Shapiro-Wilk normality test) with Tukey’s post hoc (Prism 9.0). Significance: *P < 0.05, **P < 0.01, ***P < 0.001.

## RESULTS

### NaCl-induced growth inhibition and morphometric changes

**Fig 1:**
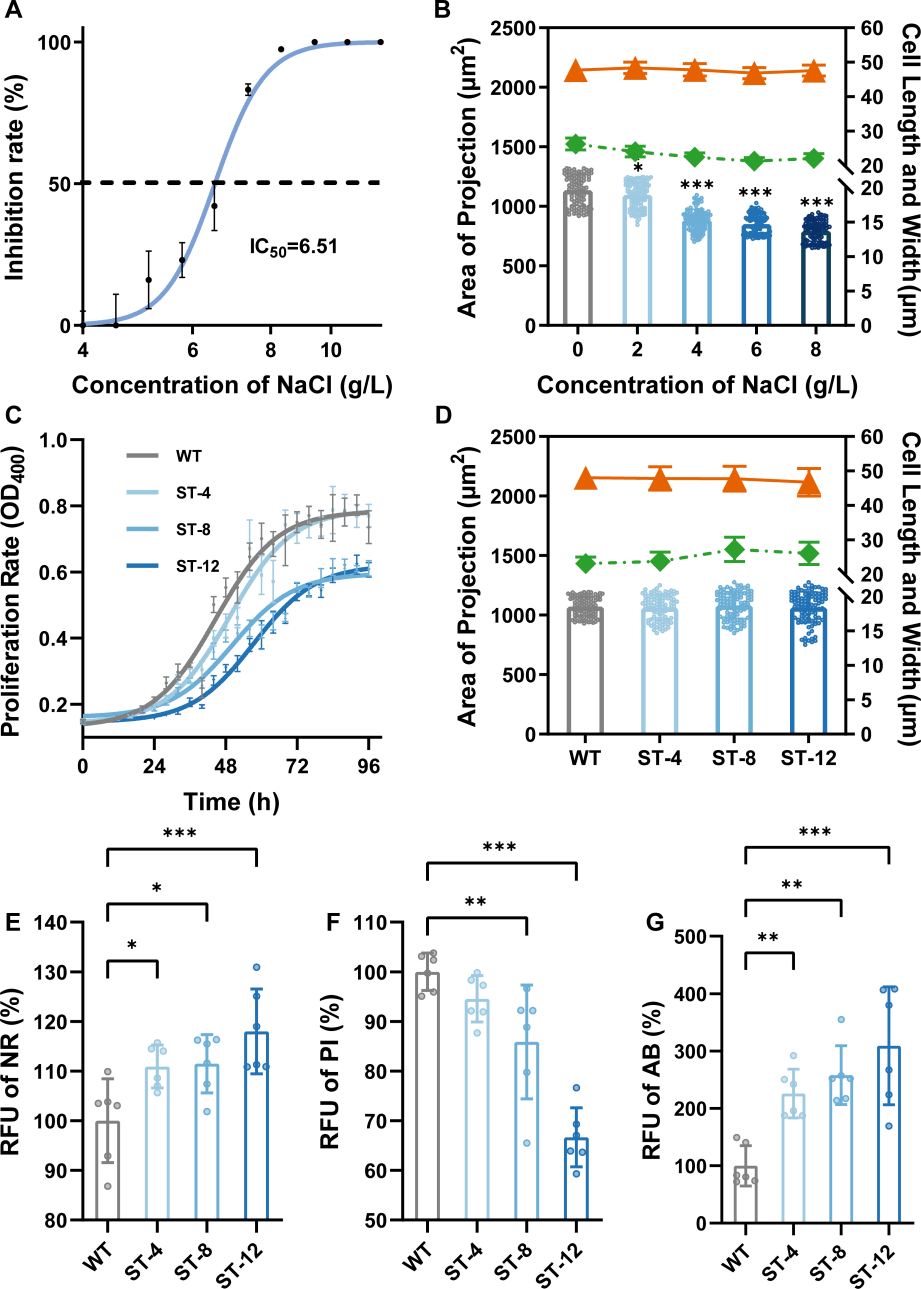
Effects of NaCl exposure on growth, cellular morphology, and viability in WT and ST strains. (**A**) Dose-dependent proliferation inhibition observed in *T. thermophila* after 24 h NaCl exposure (log10[NaCl]). (**B**) Morphometric analysis of body length (orange), body width (green), and projected cell area post 24 h NaCl exposure (mean ± SD, *n* = 100 cells/group). (**C**) Growth kinetics in WT and ST strains showing six biological replicates (*n* = 6, error bars represent SD of six parallel measurements). (**D**) Morphometric analysis of body length (orange), body width (green), and projected cell area between WT and ST strains (mean ± SD, *n* = 100 cells/strain). (**E–G**) Viability quantification using NR uptake (**E**), PI exclusion (**F**), and AB dual staining (**G**) (*n* = 6 replicates/group, three experimental repeats). Fluorescence intensities normalized to WT controls. All data expressed as mean ± SD. Statistical significance is indicated by asterisks (*P < 0.05, **P < 0.01, ***P < 0.001). In panel **B**, asterisks denote significant differences compared to the 0 g/L NaCl control group; in panels **E**, **F**, and **G**, asterisks denote significant differences between the ST strains and the corresponding WT control.

Acute NaCl exposure (24 h) dose-dependently suppressed *T. thermophila* proliferation, with complete growth arrest at 8 g/L and 95% mortality at 10 g/L (Fig. 1A). Probit analysis yielded an IC50 of 6.51 g/L NaCl (95% CI: 6.34–6.69 g/L), while sublethal doses (1–2 g/L) maintained growth kinetics comparable to controls. Analysis of the growth kinetics revealed that NaCl adaptation significantly reduced the growth rate and extended the generation time. The WT strain exhibited the fastest growth (μ = 0.046 h⁻¹, Tg = 15.1 h), while the adapted strains ST-4, ST-8, and ST-12 showed progressively slower growth and longer doubling times (see Table S3 in the Supplementary Material).

Morphometric analysis using phase-contrast microscopy revealed significant cellular shrinkage at ≥4 g/L NaCl. Compared to untreated cells (47.73 ± 1.44 μm length × 26.24 ± 1.73 μm width), 8 g/L exposure reduced dimensions to 47.56 ± 1.60 μm (length) and 22.04 ± 1.38 μm (width), representing a decrease of 21.9% (*P* < 0.001, Fig. 1B).

### Development of salt-adapted *T. thermophila* lineages

Through 3-year experimental evolution under stepwise NaCl selection pressure (4, 8, and 12 g/L), we established three clonal ST *T. thermophila* lineages (ST-4, ST-8, ST-12) exhibiting distinct growth constraints relative to WT populations. While all strains retained canonical S-shaped growth kinetics (Fig. 1C), salt-evolved lineages displayed significantly prolonged lag phases and reduced maximum cell densities (ST-12: OD_400_ = 0.63 vs WT: OD_400_ = 0.80, *P* < 0.001), despite maintaining ancestral cellular morphometrics (length: 49.5–50.8 μm, width: 25.85–29.96 μm, Fig. 1D).

Triplex fluorescent vitality profiling revealed adaptive trade-offs: the NR assay measures lysosomal activity based on the principle that only lysosomes of live cells fluoresce after accumulating the dye. The NR retention demonstrated 1.18-fold lysosomal buffering capacity in ST-12 (*P* < 0.001, Fig. 1E). We next assessed the baseline integrity of the plasma membrane in WT and ST strains under their respective maintenance conditions using PI, a dye excluded by intact membranes. Quantification of PI fluorescence revealed a constitutive, significantly lower level of signal in the ST-12 population compared to WT (a 33% reduction*, P* < 0.001, Fig. 1F). AB is a commercial preparation of resazurin dye that can be reduced to a fluorescent form by live cells, and reduced reduction indicates impaired cellular metabolism. The AB assay revealed 309% elevated metabolic activity in the ST-12 strain (*P* < 0.001, Fig. 1G). These results collectively demonstrate that chronic salt adaptation in *T. thermophila* involves metabolic reprogramming (enhanced substrate utilization efficiency) and evolutionary trade-offs under selective pressure (delayed proliferation for osmotic resilience).

### Transcriptomic profiling reveals salt-tolerant mechanisms

**Fig 2:**
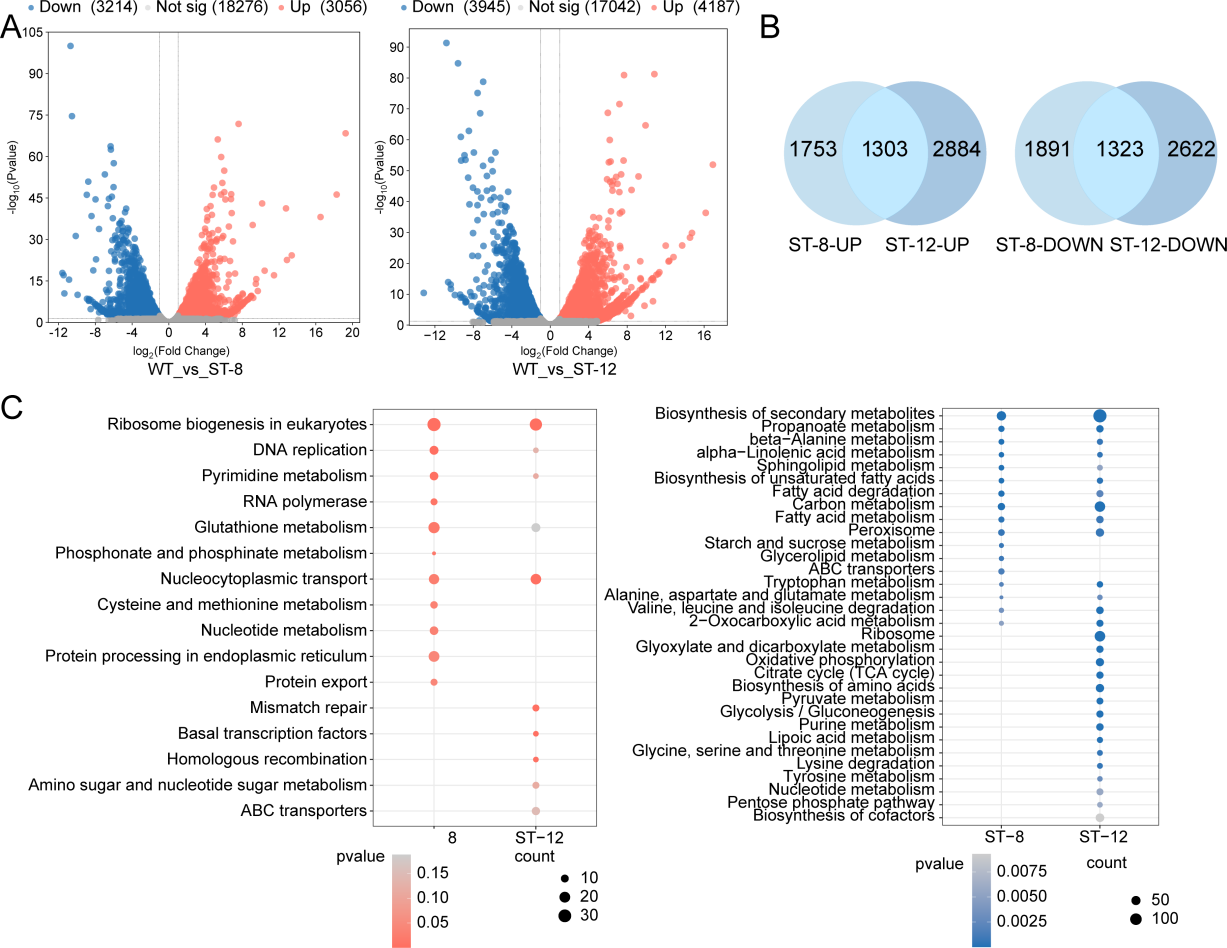
Comparative transcriptomic profiling between WT and ST-8/ST-12. (**A**) Volcano plot of DEGs in ST-8/ST-12 vs WT (|log2(fold change)| > 1, false discovery rate (FDR) < 0.05). Red: upregulated genes, blue: downregulated genes, gray: non-significant genes*.* (**B**) Number of genes commonly upregulated and downregulated by ST-8/ST-12*.* (**C**) KEGG enrichment analysis of DEGs between ST-8 and ST-12. Red/blue circles represent pathways enriched in upregulated/downregulated genes, respectively. Circle area correlates with gene count, color intensity *P* value*.*

RNA sequencing of WT and ST *T. thermophila* strains yielded 409 million high-quality paired-end reads (Q30 > 91.98%, Table S4). Principal component analysis demonstrated significant transcriptional divergence between ST-8 and ST-12 lineages, with the first principal component (PC1) explaining 57.21% of the total variance (Fig. S1A). Comparative analysis identified 6,270 DEGs in ST-8 (3,056↑/3,214↓) and 8,132 DEGs in ST-12 (4,187↑/3,945↓), including 1,303 shared upregulated and 1,323 co-downregulated genes (Fig. 2A and B). For co-upregulated genes, ribosome biogenesis in eukaryotes (tet03008) was the most significantly enriched pathway in both ST-8 and ST-12. In ST-8, this pathway contained 32 enriched genes with a *P* value of 2.94 × 10⁻¹⁰; in ST-12, it had 27 enriched genes and a *P* value of 2.67 × 10⁻¹⁰, demonstrating sustained activation of ribosome synthesis in strains adapted to both NaCl concentrations. ST-8 showed significant enrichment (*P* < 0.05) of 10 additional pathways, including DNA replication (tet03030) (13 genes*, P* = 0.00)*,* pyrimidine metabolism (tet00240) (12 genes*, P* = 0.00), glutathione metabolism (tet00480) (22 genes*, P* = 0.02), and protein processing in endoplasmic reticulum (tet04141) (20 genes*, P* = 0.04), indicating active DNA replication, enhanced antioxidant defense, and efficient protein quality control in ST-8. In contrast, ST-12 exhibited a distinct enrichment profile: nucleocytoplasmic transport (tet03013) (19 genes*, P* = 0.00), mismatch repair (tet03430) (8 genes*, P* = 0.00), and homologous recombination (tet03440) (6 genes*, P* = 0.00) were significantly enriched. It is noteworthy that pathways enriched in ST-8, including DNA replication (tet03030) (6 genes*, P* = 0.13), glutathione metabolism (tet00480) (13 genes*, P* = 0.19), and pyrimidine metabolism (tet00240) (6 genes*, P* = 0.11), exhibited reduced significance (*P* > 0.10) in ST-12.

In order to verify the accuracy and reliability of transcriptome sequencing, nine DEGs involved in redox reactions, lipid metabolism, and DNA replication were randomly selected and analyzed by qRT-PCR. qRT-PCR correlation with RNA-seq was performed using a scatterplot with log2-fold variation, and both techniques showed positive correlation coefficients (Pearson’s coefficient R^2^ = 0.98), which demonstrated the sequencing data reliability (Fig. S3).

### Proteomic profiling confirms salt-tolerant mechanisms

**Fig 3:**
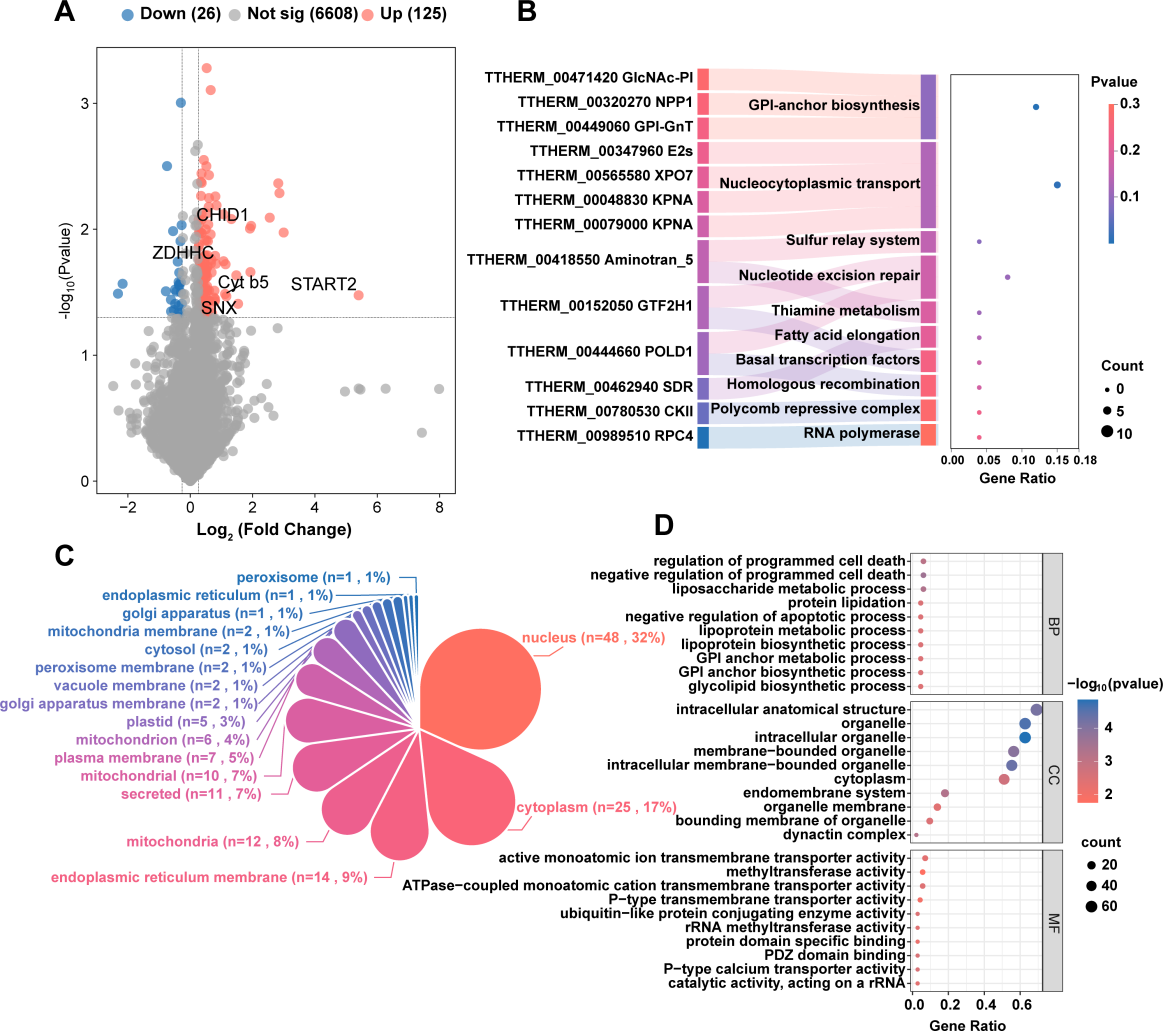
Comparative proteomic profiling between WT and ST-8. (**A**) Volcano plot analysis of DAPs between ST-8 and WT groups. Proteins with significant changes (|fold change| ≥ 1.2, *P* < 0.05) are highlighted in red (upregulated) and blue (downregulated). Gray dots represent proteins without significant alteration. (**B**) KEGG pathway enrichment analysis of DAPs. Top 10 significantly enriched pathways (*P* < 0.05) are displayed with bubble size indicating the number of mapped proteins and color intensity representing the enrichment significance (*P* value). (**C**) Subcellular localization distribution of DAPs. Pie chart segments represent percentage proportions categorized using the PSORT subcellular localization prediction system. (**D**) Gene Ontology (GO) classification of DAPs. The horizontal axis indicates enrichment ratio (number of DAPs per term divided by total annotated proteins in that term), while the vertical axis displays three GO categories: biological process (BP), molecular function (MF), and cellular component (CC). Significance threshold was set at *P* < 0.05 by Fisher’s exact test with Benjamini-Hochberg correction.

Comparative proteomic analysis of WT and ST-8 strains identified 6,759 high-confidence proteins following stringent data preprocessing (FDR < 1%). Differential abundance profiling (fold change ≥1.2 or ≤0.83*, P* < 0.05 by moderated *t*-test) revealed 125 significantly upregulated and 26 downregulated proteins in ST-8, visualized via volcano plot analysis (red: upregulated, blue: downregulated, Fig. 3A). Subcellular localization mapping demonstrated that differentially abundant proteins (DAPs) predominantly localize to functional hotspots: nucleus (27.2% of DAPs), cytoplasm (22.4%), endoplasmic reticulum (ER) membranes (18.1%), mitochondria (15.3%), and secretory pathways (10.6%) (Fig. 3C). Among the most prominently upregulated proteins were lipid metabolism-associated START2 (UniProt: Q22BV5, TTHERM_01084120, 42.3-fold), chitinase domain-containing protein CHID (I7MLK3, TTHERM_00729150, 3.9-fold), sorting nexin SNX (I7M7V2, TTHERM_00127270, 2.9-fold), steroid-binding cytochrome b5 (I7MEQ5, TTHERM_00338510, 2.2-fold), and palmitoyltransferase ZDHHC (Q23DT9, TTHERM_00045030, 1.8-fold), collectively suggesting enhanced lipid remodeling and membrane stabilization (Fig. 3B).

KEGG pathway enrichment analysis (*P* < 0.05) identified GPI-anchor biosynthesis (tet00563, *P* = 0.004) and nucleocytoplasmic transport (tet03013, *P* = 0.01) as central adaptive mechanisms (Fig. 3B), aligning with GO term analysis showing significant enrichment in protein lipidation (GO:0006497*, P* = 0.003) and glycolipid biosynthetic processes (GO:0009247*, P* = 0.004), which collectively support membrane stabilization under ionic stress (Fig. 3D). Intriguingly, coordinated negative regulation of apoptotic processes (GO:0043069*, P* = 2.6 × 10⁻⁴) and suppression of programmed cell death (GO:0043066*, P* = 0.003) suggest enhanced survival prioritization, while upregulated liposaccharide metabolic processes (GO:1903509*, P* = 5 × 10⁻^4^) and lipoprotein biosynthesis (GO:0042158*, P* = 0.003) indicate lipidome remodeling for osmotic homeostasis.

At the cellular component level, DAPs were significantly enriched in organelle membranes (GO:0031090*, P* = 0.004), especially in the endomembrane system (GO:0012505*, P* = 5.4 × 10⁻⁴) and the dynactin complex (GO:0005869*, P* = 5.8 × 10⁻⁴), showing structural adaptability in vesicle trafficking and cytoskeleton organization. At the molecular function level, differentially expressed proteins were enriched in P-type calcium transporter activity (GO:0005388*, P* = 0.003) and ATPase-coupled cation transport (GO:0019829*, P* = 0.004), as well as ubiquitin-like protein binding activity for protein quality control (GO:0061650*, P* = 0.003), indicating that protein turnover was enhanced to adapt to high salt (Fig. 3D).

### Metabolic reprogramming and the redox paradox

**Fig 4:**
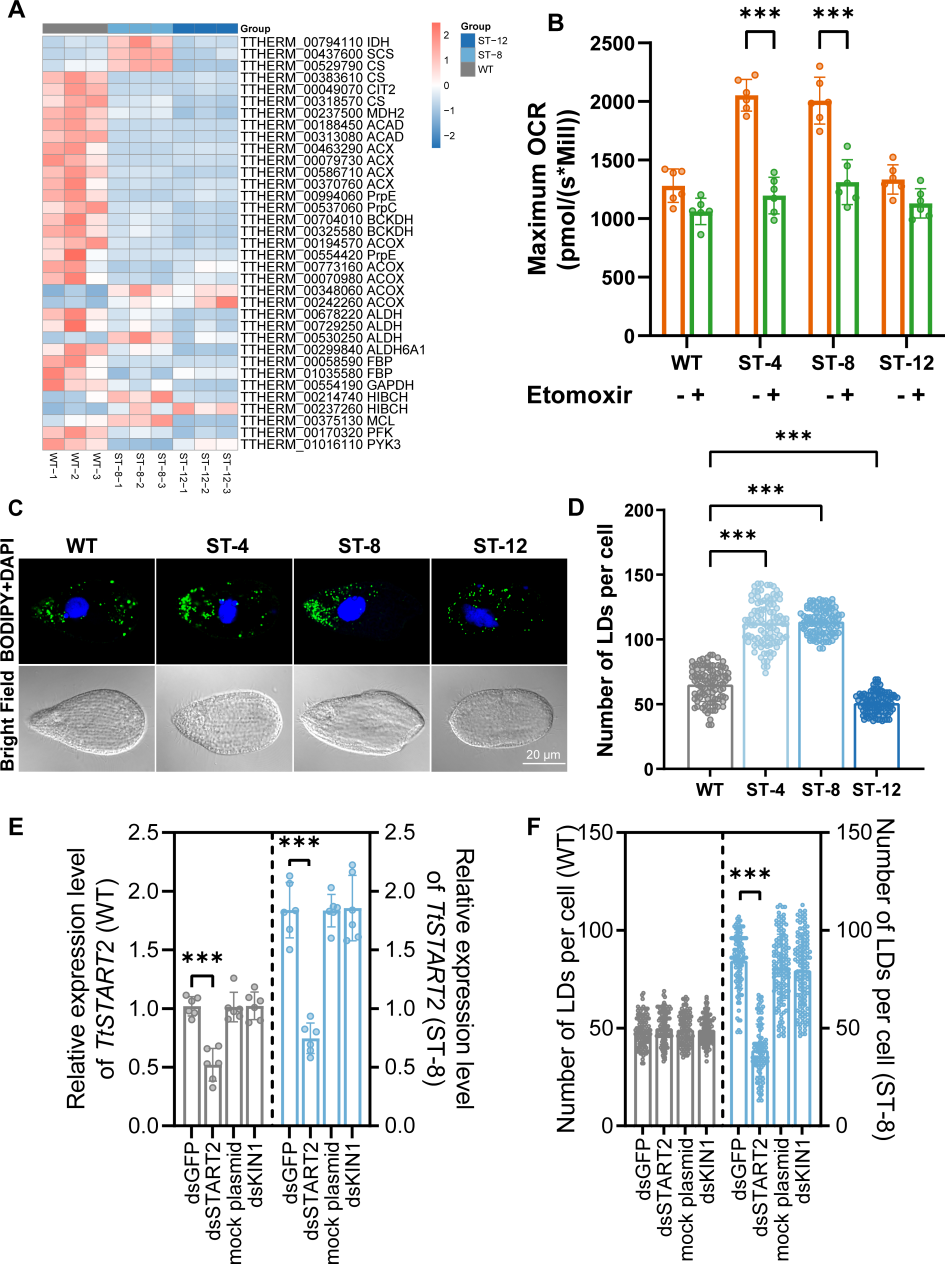
Functional characterization of metabolic alterations in WT and ST strains. (**A**) Heatmap visualization of DEGs associated with glycolysis/gluconeogenesis, the TCA cycle, and propionic acid metabolism in ST-8 vs ST-12 strain. Key enzyme abbreviations: IDH, isocitrate dehydrogenase; SCS, succinyl-CoA synthetase; CS, citrate synthase; CIT2, CITrate synthase 2; MDH2, malate dehydrogenase 2; ACX, peroxisomal acyl-CoA oxidase; ACAD, acyl-CoA dehydrogenase; PrpE, propionyl-CoA synthetase; PrpC, 2-methylcitrate synthase/citrate synthase II; ACOX, acyl-coenzyme A oxidase; FBP, fructose-1,6-bisphosphatase; PFK, 6-phosphofructokinase; BCKDH, branched-chain α-keto acid dehydrogenase; ALDH, aldehyde dehydrogenase; ALDH6A1, methylmalonate-semialdehyde dehydrogenase; GAPDH, glyceraldehyde-3-phosphate dehydrogenase; HIBCH, 3-hydroxyisobutyryl-CoA hydrolase; MCL, methylisocitrate lyase; PYK3, PYruvate kinase. (**B**) Dynamic changes in maximal OCR of WT and ST strains following treatment with 25 μM etomoxir (mitochondrial fatty acid oxidation inhibitor). Data represent mean ± SD, **P < 0.01, ***P < 0.001 vs untreated control. (**C**) Representative confocal microscopy images of BODIPY 493/503-stained lipid droplets (green) in WT and ST strains. Nuclei were counterstained with DAPI (blue). Scale bar: 20 μm. (**D**) Quantitative analysis of lipid droplet (LD) density per cell in WT and ST strains (*n* ≥ 90 cells per group). Data presented as mean ± SD, ***P < 0.001. (**E**) Relative mRNA expression levels of TtSTART2 in WT and ST-8 cell lines 24 h post-RNA interference (siRNA-mediated knockdown). Expression normalized to 17S (housekeeping gene), ***P < 0.001 vs siRNA-GFP control (one-way ANOVA). (**F**) Alterations in lipid droplet abundance in WT and ST-8 cells following TtSTART2 knockdown (*n* ≥ 90 cells per group). Data shown as mean ± SD, ***P < 0.001 (two-tailed Mann-Whitney U test).

ST exhibited a marked redox imbalance characterized by a surge in reactive oxygen species (ROS) (2.8–4.1-fold increase relative to WT*, P* < 0.001), accompanied by glutathione (GSH) depletion (Δ20%–33%*, P* < 0.01), despite stable malondialdehyde levels (Fig. S5A through C). This oxidative stress signature was associated with a decoupling of glutathione S-transferase (GST) regulation, with transcriptional upregulation contrasting with a 50% loss of enzyme activity (*P* < 0.001). Concomitant inhibition of superoxide dismutase (SOD) activity (16%–45% decrease*, P* < 0.02) was observed, while catalase (CAT) activity remained unchanged compared to WT. O_2_K analysis revealed a lipid-centric reprogramming that was primarily fatty acid-dependent, with etomoxir (CPT1 inhibitor) reducing mitochondrial oxygen consumption rate (OCR) by 35%–42% (*P* < 0.005), while inhibition of glycolysis (UK5099) or glutaminolysis (BPTES) had no effect (Fig. 4B; Fig. S4D). Transcriptional shifts were reflected in the suppression of glycolytic genes (HK↓4.7-log2FC, PFK↓3.2-log2FC) and the upregulation of the tricarboxylic acid (TCA) cycle (CS↑2.3-log2FC, IDH↑1.8-log2FC) (Fig. 4A). The paradox of lactate production, where lactate accumulated despite inhibition of glycolysis (2.1-fold↑*, P* < 0.001), implies alternative NAD^+^ regeneration, possibly through activation of the malate-aspartate shuttle or alanine transamination (ST-4 pyruvate↑1.2-fold vs ST-12↓1.7-fold, Fig. S6F).

Lipidomic adaptations were central to salt tolerance. Intracellular lipid droplets increased 1.7-fold in ST-4 (*P* < 0.001) and 1.7-fold in ST-8 (*P* < 0.001) (Fig. 4C and D), coinciding with reduced lipase activity (−37% to 94%*, P* < 0.001) but stable triacylglycerol levels (Fig. S4E). The efficacy and specificity of the RNAi knockdown were confirmed. First, qRT-PCR verified a significant reduction in *START2* transcript levels in both WT and ST-8 strains following RNAi treatment (Fig. 4E). Critically, the negative controls—including RNAi targeting the exogenous GFP, the endogenous non-related *KIN* gene, and the empty L4440 vector—all showed no significant effect on lipid droplet abundance compared to the untreated control in both WT and ST-8 strains (Fig. 4E and F), thereby validating the specificity of the *START2* knockdown phenotype. Functional validation via RNAi knockdown of the lipid-associated START2 gene (TTHERM_01084120, 42.3-fold upregulated in proteomics) specifically reduced lipid droplets in ST-8 (−54%*, P* < 0.001) without affecting WT (Fig. 4E and F). This demonstrates that salt adaptation requires START2-mediated lipid storage optimization, independent of canonical lipolysis pathways.

### DNA replication stress underlies genomic instability in ST *T. thermophila*

**Fig 5:**
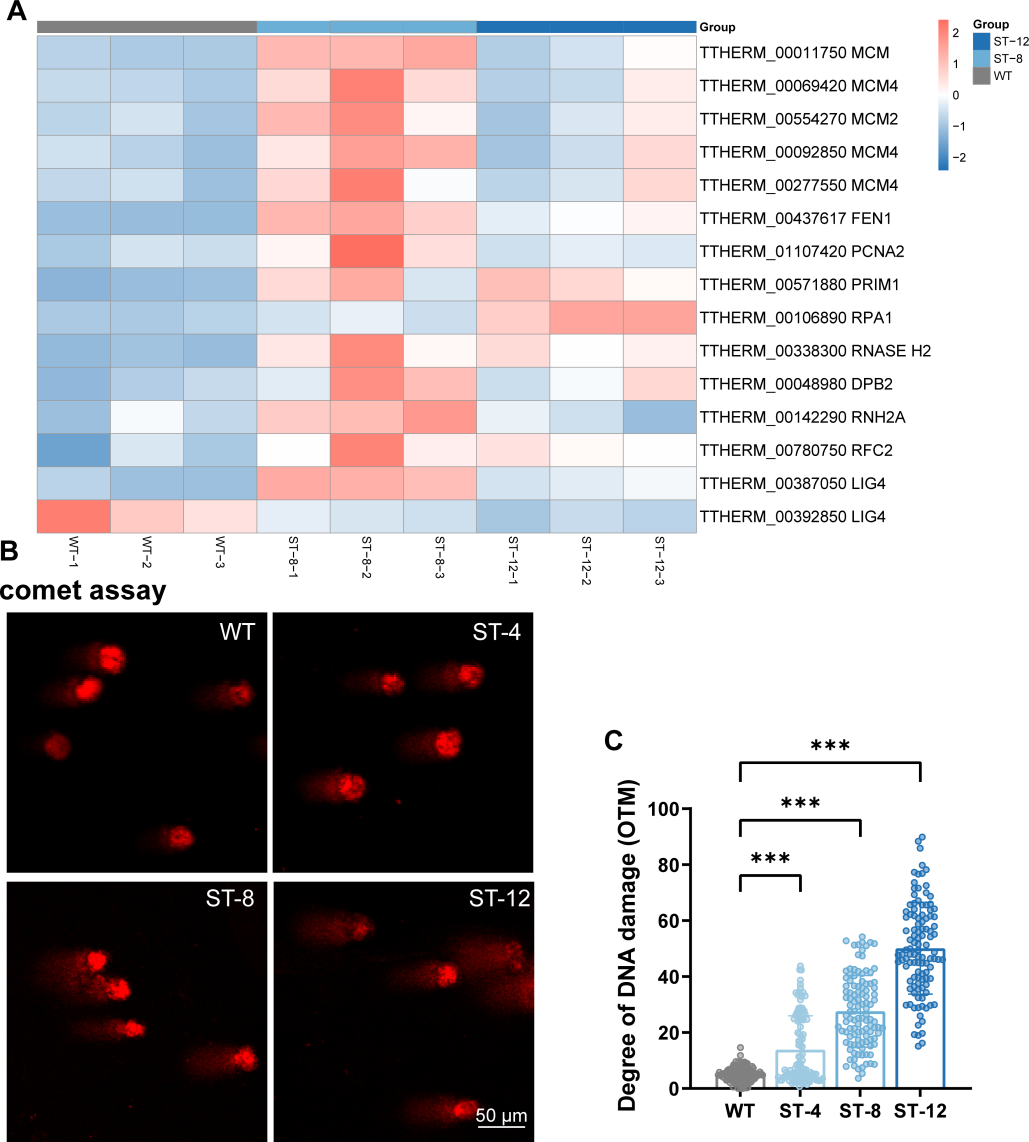
DNA replication fidelity and damage in WT and ST strains. (**A**) Hierarchical clustering heatmap of expression changes for core DNA replication genes across WT, ST-8, and ST-12. Gene abbreviations with full nomenclature: RPA1, replication protein A1; FEN1, flap endonuclease 1; PRIM1, DNA primase subunit 1; RNASE H2, ribonuclease H2 subunit A; RFC2, replication factor C subunit 2; RNH2A, ribonuclease H2 catalytic subunit; PCNA, proliferating cell nuclear antigen; MCM2-7, minichromosome maintenance complex 2-7; DPB2, DNA polymerase ε subunit B. Color scale: red (upregulated), blue (downregulated), significance threshold: |log2(fold change)| ≥1, FDR-adjusted *P* < 0.05. (**B and C**) Quantitative assessment of DNA damage via alkaline comet assay. (**B**) Representative fluorescence micrographs of PI-stained nuclei showing comet tails (DNA fragmentation) under 40× magnification. Scale bar: 50 μm. (**C**) DNA damage index quantified by Olive tail moment (*n* > 100 cells per group). Data presented as median ± interquartile range, ***P < 0.001 vs WT.

Transcriptomic and proteomic analyses revealed that ST *T. thermophila* imposes significant replication stress, characterized by dysregulation of core DNA replication machinery. Key replisome components involved in replication initiation, including PRIM1 (DNA primase catalytic subunit, 2.4-fold upregulation*, P* < 0.001), were markedly elevated in ST strains, alongside elongation factors RPA (replication protein A, 1.2–3.0-fold) (Fig. 5A). Processing enzymes RFC2 (clamp loader subunits, 1.3–1.6-log2FoldChange) and FEN1 (flap endonuclease, 1.1–1.7-log2FoldChange)—were significantly upregulated in ST strains (Fig. 5A). Furthermore, core DNA repair machinery also showed strong regulation. The key DNA ligase LIG4 (TTHERM_00387050) was significantly upregulated (2.78–4.30-log2FoldChange), indicating an active role in resolving replication stress. Interestingly, a putative paralog, TTHERM_00392850, exhibited a significant opposite trend, showing notable downregulation (−1.72 to −1.26 log2FoldChange) (Fig. 5A).

Consistent with this hypothesis, alkaline comet assays demonstrated dose-dependent genomic damage across ST lineages. All ST strains exhibited significantly elevated OTM values compared to WT (ST-4: 13.9 ± 5.4, ST-8: 27.6 ± 13.8, ST-12: 50.14 ± 27.6, *P* < 0.001 vs WT 5.0 ± 1.6), with ST-12 showing near 10-fold greater damage (Fig. 5B and C). The concurrent upregulation of RPA and PRIM1 suggests a compensatory mechanism to stabilize stalled forks under ionic interference. Ultimately, this adaptive strategy tolerates replication-derived genomic instability as a trade-off for survival under chronic salt stress, prioritizing rapid replication restart and osmotic homeostasis over perfect genome maintenance.

### Mitochondrial plasticity under salt

We first sought to distinguish the stable mitochondrial adaptations in ST strains from acute stress responses in WT cells. As shown in Fig. S7A through F, short-term (24 h) exposure of WT cells to sub-lethal salt stress (4 and 8 g/L NaCl) triggered a pathological mitochondrial state. This was characterized by significant organelle swelling and elongation (Fig. S7E and F) and a severe impairment of oxidative phosphorylation coupling, evidenced by a marked reduction in the RCR (Fig. S7D). In contrast, the activities of individual electron transport chain complexes (CI, CII, CIV) remained largely unchanged (Fig. S7A through C), highlighting that the acute stress specifically disrupts the coordinated function of the respiratory system rather than the individual components.

Having established the dysfunctional nature of the acute response, we then validated the stability of the long-term adapted state. A critical test was to challenge the strains with lethal salt concentrations beyond their adaptation levels. As demonstrated in Fig. S7G, WT cells failed to survive at 16 and 18 g/L NaCl. In stark contrast, the ST strains exhibited a dose-dependent acquisition of extreme tolerance, with ST-8 and ST-12 maintaining viability under these lethal conditions. This graded survival fidelity, which correlates with their acclimation history, provides conclusive evidence for a stable, long-term adaptive mechanism, as opposed to a transient stress response. Against this backdrop, the mitochondrial phenotype of the stably adapted ST strains represents a refined, functional reprogramming rather than a stress-induced pathology.

**Fig 6:**
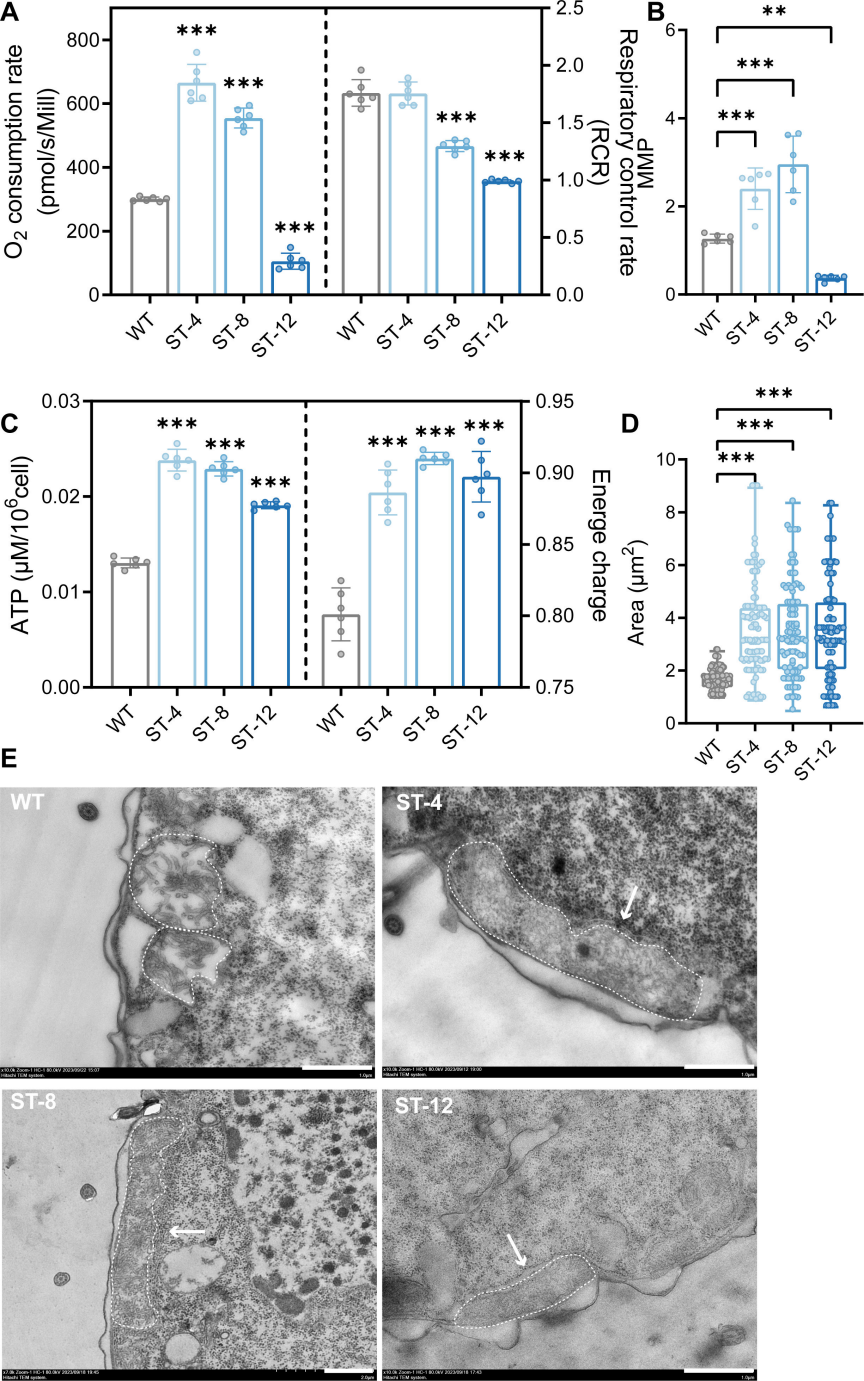
Mitochondrial functional and structural remodeling in WT and ST strains. (**A**) Comparative analysis of mitochondrial complex II activity and RCR. Left: Complex II (succinate dehydrogenase) activity measured by O2K (*n* = 6 biological replicates). Right: RCR calculated as State 3/State 4 respiration rates using O2K (*n* = 6). Data expressed as mean ± SD, ***P < 0.001 (unpaired Student’s *t*-test). (**B**) MMP quantification via JC-1 staining. Red/green fluorescence ratio (aggregates/monomers) analyzed by **P < 0.01, ***P < 0.001 vs WT. (**C**) Bioenergetic profiling. Left: cellular ATP levels measured by HPLC. Right: energy charge (EC) calculated as (ATP + 0.5 ADP)/(ATP + ADP + AMP). Data normalized to cell number (*n* = 6), ***P < 0.001 (one-way ANOVA with Bonferroni correction). (**D**) Morphometric analysis of mitochondrial area. Representative TEM images analyzed by ImageJ (*n* = 100 mitochondria per group). ***P < 0.001. (**E**) Ultrastructural characterization by TEM. White arrows: ER-mitochondria contact sites (MERCs). mt: mitochondria with cristae remodeling. Scale bar: 1 μm.

The activity of mitochondrial complexes in WT and ST cells revealed different electron transport chain remodeling. Complex II (CII) was overactivated: succinate dehydrogenase activity surged 2.1-fold in ST-4 (*P* < 0.001) and 1.5-fold in ST-8 (*P* < 0.01), but plummeted to 50% WT level in ST-12 (Fig. 6A), indicating threshold-dependent saturation of FADH2 utilization. Complex IV (CIV) was biphasically regulated: cytochrome c oxidase activity increased modestly in ST-4/ST-8 (1.3–1.5-fold), while ST-12 showed 60% inhibition (*P* = 0.09), which was consistent with respiratory uncoupling (RCR ↓48%*, P* < 0.001) (Fig. 6A; Fig. S6C).

Concurrently, mitochondrial bioenergetics shifted toward ATP-centric adaptation. MMP in ST-4 increased 1.8-fold (*P* < 0.001), associated with ATP overproduction (+0.7-fold) despite reduced oxygen flux (Fig. 6B and C). EC shifted from 0.8 (WT) to 0.9 (*P* < 0.001), indicating a dominance of catabolism over biosynthesis (Fig. 6C). Mitochondrial area in ST increased 2.2-fold (3.45–3.64 vs 1.60 μm²*, P* < 0.001), and endoplasmic reticulum-mitochondria contact sites (MERCs) were abundant (Fig. 6D and E), which may facilitate lipid transfer for β-oxidation.

## DISCUSSION

A comprehensive analysis reveals that long-term salt adaptation in *T. thermophila* is a multifaceted process achieved through profound cellular reprogramming. The evolved strains exhibit a suite of complex adaptive traits centered on membrane stabilization, metabolic rearrangement, organelle restructuring, and managed trade-offs. When compared with other eukaryotes ranging from plants to mammals, these mechanisms show fascinating similarities and singularities, highlighting both conserved principles and unique innovations in coping with ionic stress.

### Membrane remodeling, lipid storage, and ion homeostasis

A cornerstone of salt adaptation across species is the remodeling of cell membranes to maintain fluidity and integrity under osmotic and ionic stress (45). In plants and yeast, this typically involves altering phospholipid headgroup and sterol composition (46, 47). Our proteomic data are consistent with this principle but reveal a unique molecular focus in *T. thermophila*. The significant upregulation of the lipid transfer protein START2 and its functional validation by RNAi suggest that the storage of neutral lipids within lipid droplets is crucial for salt tolerance (Fig. 4E and F) (48, 49). The observation that START2 knockdown specifically reduced lipid droplets in the adapted ST-8 strain, but not in the WT, underscores its context-dependent essentiality: it becomes indispensable only after the cell has undergone the lipid-centric adaptive rewiring necessary for salt tolerance. This strategy parallels observations in the salt-tolerant alga *Dunaliella salina*, where lipid droplets serve as both energy reservoirs and protective stores of fatty acids to mitigate lipotoxicity under stress (50). However, the central role of a START family protein in this process is novel. While the START domain is known to be responsible for lipid binding in mammals (51), its direct role in mediating environmental stress tolerance through lipid droplet biogenesis is less well documented (52). The simultaneous enrichment of GPI-anchor biosynthesis and protein lipidation pathways further emphasizes this as a comprehensive membrane-centric adaptation strategy (Fig. 3B) (53), reminiscent of the enhanced membrane repair mechanisms in salt-adapted Saccharomyces cerevisiae (54). This membrane and lipid remodeling is complemented by a conserved emphasis on ion homeostasis, exemplified by the upregulation of V-type ATPases and other transmembrane transporters, a common theme in ciliates like *Euplotes crassus* and *Uronema marinum* (55). Thus, while *T. thermophila* employs the conserved strategies of membrane/lipid remodeling and ion transport, it utilizes a unique set of molecular executors, among which START2 emerges as a key protagonist for lipid storage.

### Mitochondrial plasticity and enhanced MERCs for integrated stress response

One of the most striking morphological adaptations of the ST strain is the significant expansion of mitochondrial area and the enrichment of mitochondrial-endoplasmic reticulum contact sites (Fig. 6D and E). This is not a passive swelling but an active structural reorganization that is likely central to physiological adaptation (56–58). Mitochondrial enlargement, often associated with enhanced fusion or impaired fission, is a known response to various stresses, acting to dilute oxidative damage and improve metabolic efficiency, as seen in nutrient-starved mammalian cells (59–63). In our ST strains, this mitochondrial expansion is coupled with a functional shift toward fatty acid oxidation (Fig. 4B), forming a specialized compartment for efficient energy extraction from lipids.

Crucially, the proliferation of MERCs provides a structural basis for the observed metabolic and lipid adaptations. MERCs are important hubs for lipid transfer (64), calcium signaling (65), and apoptosis regulation (66). The notion that MERCs are dynamic, metabolically responsive platforms is reinforced by a study on VAPB (64), which provides a direct structural parallel to our observation of MERC proliferation in *T. thermophila*. This congruence indicates that the salt-adapted ciliate exhibits a sophisticated, dynamic inter-organelle connectivity. The enrichment of lipid metabolism-related proteins in the ER and the dependence of mitochondria on fatty acid oxidation suggest that these contact sites are promoting the rapid transfer of lipids*,* perhaps as phospholipid precursors or fatty acids for β-oxidation (67). This echoes findings in hepatocytes, where enhanced MERC activity promotes lipid exchange and mitochondrial respiration (68). Furthermore, inositol 1,4,5-trisphosphate receptors (IP3Rs) at MERCs are critical for calcium shuttling, with the IP3R subtype recently implicated in a structural role for sustaining ER-mitochondria contact sites and fine-tuning local calcium microdomains (69, 70). The stable MMP and ATP overproduction observed in our ST-4/ST-8 strains (Fig. 6A through C), despite respiratory impairment, may be facilitated by the optimized calcium-mediated allosteric regulation of key dehydrogenases at these organelle interfaces (71). The significance of such organellar connectivity is underscored by its conservation; for instance, a 2024 study revealed that LBR protein acts as a tether to build mitotic ER-mitochondria contacts, which boost mitochondrial Ca²^+^ influx and ATP production to power cell division (71). Similarly, MERCs’ expansion and associated Ca²^+^ transfer enhancement during mitosis have been reported to support increased energy demands (72). Consequently, the restructuring of mitochondrial architecture and its tight physical coupling with the ER represent a refined, organelle-level adaptation that integrates energy production, lipid trafficking, and stress signaling—a mechanism increasingly recognized as pivotal in metazoan stress responses and now highlighted in a unicellular context.

### Metabolic and redox reprogramming

The widespread transcriptional repression of core energy metabolism pathways (Fig. 4A)*,* particularly the significant metabolic downregulation in the highly tolerant ST-12 strain, is likely a survival strategy selected by evolution under chronic salt stress. However, our findings reveal a subtle metabolic rearrangement occurring within this repressed background. The shift to fatty acid-dependent respiration, which can be inhibited by etomoxir, suggests that cells preferentially utilize lipids for efficient ATP production, a metabolic signature shared by certain cancer cells and starving mammals (73–75). At the same time, the paradoxical phenomenon of lactate accumulation occurring simultaneously with glycolysis inhibition suggests a redirection of carbon flow (Fig. S6F)*,* possibly through the pyruvate-alanine cycle or the malate-aspartate shuttle, mechanisms that are crucial for maintaining redox balance in mammalian mitochondria under hypoxic conditions (76, 77). This suggests that ST strains restructure their metabolic networks not only to conserve energy but also to maintain redox homeostasis, even at the expense of falling into an apparent dysregulated state characterized by a surge in ROS and depletion of GSH. This “redox paradox” reflects the concept of “oxidative signaling” in plants, whereby specific ROS waves act as second messengers to coordinate stress responses, rather than simply toxic byproducts (78, 79). Our data extend this concept to salt-adapted Tetrahymena: the concerted inhibition of key antioxidants (SOD, GST) and absence of CAT compensation, despite high ROS and successful proliferation (Fig. S5A through C), imply that H₂O₂ is tolerated as a signaling molecule. This suggests a managed oxidative state where H₂O₂-mediated signaling is prioritized over its complete detoxification (80).

The dissociation of GST transcription and activity indicates the presence of a sophisticated post-transcriptional regulatory layer within the antioxidant system (Fig. S5C and D). This phenomenon has been observed in *Alexandrium pacificum* in response to environmental pollutants, such as NaOCl and PCBs (81). Concurrently, the initial overactivation of mitochondrial Complex II (succinate dehydrogenase) aligns with findings in *Caenorhabditis elegans* under oxidative stress, where the modulation of its activity serves to manage electron flow and ROS production (82).

### Genomic trade-offs and adaptive costs under replication stress

The transcriptomics data demonstrate that ST *T. Tetrahymena* respond to osmotic stress challenges by actively inducing replication stress, a process accompanied by significant genomic instability. There is also evidence to suggest that cells may maintain survival under salt stress by promoting replication restart rather than strict fidelity, as indicated by the synergistic up-regulation of key replication initiation and elongation factors (e.g.*,* PRIM1, RPA, and FEN1) (Fig. 5A). The existence of this adaptive cost is further supported by the dose-dependent DNA damage observed in the comet assay (Fig. 5B). This phenomenon has been demonstrated in a variety of biological systems. For instance, in cancer cells, oncogene-induced replicative stress is a pivotal catalyst of genomic instability and tumor evolution (83). In bacterial systems, stressful environments trigger analogous mechanisms that accelerate evolutionary adaptation by increasing mutation rates (84). It is noteworthy that DNA damage repair systems are frequently initiated in response to replication stress when *T. thermophila* is subjected to additional stresses. For instance, the RNA interference pathway in Tetrahymena has been demonstrated to safeguard genome integrity through the action of specific small RNAs (85). Furthermore, it has been demonstrated that defects in histone H3 lysine 27 monomethylation (H3K27me1) have the capacity to impede replication elongation and to induce replicative stress by affecting PCNA (86). This resource reallocation is further evidenced by the DNA ligase regulation: LIG4 is strongly induced for repair, while the related ligase TTHERM_00392850 is simultaneously suppressed (Fig. 5A). ST strains may have adopted a unique trade-off strategy: tolerating replication-associated errors to a certain extent, and reallocating energy and resources that would otherwise be used for high-fidelity repair to imminent osmoregulatory processes. This “prioritization of survival over genomic fidelity” strategy is analogous to the stress response of bacteria and the rapid proliferation of cancer (87, 88), and creates a permissive environment for the accumulation of mutations under long-term stresses.

### A comparative perspective on stress adaptation in *T. thermophila*

A comparative analysis with other stress models in *T. thermophila*, such as adaptation to Pb(II) or exposure to CuO nanotubes, reveals a core set of conserved defense mechanisms deployed against diverse insults. Notably, the significant upregulation of START domain proteins in our salt-adapted strains finds a direct parallel in Pb(II)-adapted cells, underscoring a universal role for lipid storage and trafficking in cellular defense (89). This is further supported by the common induction of lipid droplets under both salt and nanomaterial stress (90, 91)*,* positioning them as key multifunctional organelles in general stress management. Beyond lipids, shared enhancements in the ubiquitin-proteasome system for protein quality control, alongside upregulation of dynamitin and P-type ATPases for maintaining cytoskeletal and ionic homeostasis, suggest that *T. thermophila* employs a common toolkit to preserve core cellular functions under various abiotic threats (89, 92–96). This concept of a shared defense arsenal is further exemplified by the oxidative stress response. The transcriptional upregulation of specific GST genes (e.g., TthGSTO1, TthGSTM6) in our salt-adapted strains aligns with their induction under heavy metal stress (97, 98), marking them as components of a core detoxification toolkit. However, the concomitant overall loss of GST enzyme activity in our strains points to a stressor-specific functional impairment, suggesting that salinity uniquely disrupts the post-transcriptional implementation of this otherwise conserved response.

Despite the presence of these shared strategies, long-term salt adaptation instigates a unique and profound physiological restructuring. The extreme mitochondrial reprogramming—characterized by a shift to fatty acid oxidation, massive organelle expansion, and proliferation of MERCs—forms a distinctive bioenergetic adaptation, likely tailored to the relentless demands of osmoregulation. Furthermore, the observed tolerance for replication stress and associated genomic instability represents a high-stakes trade-off not prominently featured in other stress responses, indicating that survival under chronic osmotic pressure may uniquely prioritize cellular continuity over genomic fidelity.

In summary, salt adaptation in *T. thermophila* is not an isolated phenomenon but is built upon a foundation of conserved stress response pathways*,* particularly in lipid and protein homeostasis. However, the unique selective pressure of salinity fine-tunes this common toolkit, driving a distinctive adaptive signature defined by specialized mitochondrial function and a singular trade-off with genome stability.

### Conclusion

This study demonstrates that the survival of protists under chronic salinity depends on the evolutionary synergy among osmoregulation, metabolic reprogramming, and genomic adaptation. *T. thermophila* achieves osmoregulation by coordinating LD storage and adjusting ion channel proteins. ST strains have been shown to utilize controlled genomic instability as an evolutionary catalyst, balancing replication stress-induced mutagenesis with DNA repair optimization to accelerate adaptation. Mitochondrial reorganization prioritizes energy production efficiency over respiratory rate by enhancing contact with the endoplasmic reticulum. Paradoxically, these organisms circumvent typical antioxidant systems by compartmentalizing oxidative damage and redirecting metabolic resources toward osmoprotectant synthesis, a trade-off that maintains redox balance at the expense of enzymatic defense capacity.

The discovery that the expansion of mitochondrial-endoplasmic reticulum contacts and the accumulation of LDs are universal characteristics of eukaryotic salt tolerance offers a predictive framework for assessing microbial responses to environmental salinization. These findings have prompted a re-evaluation of the theory of extreme microbial adaptation, emphasizing the capacity of unicellular eukaryotes to integrate prokaryotic osmoregulatory strategies with organelle-level coordination. This approach has significant implications for the development of stress-resistant microorganisms and the prediction of collapse thresholds in aquatic ecosystems.

### Highlights

1. Three-year experimental evolution delineates *T. thermophila* adaptation to 4–12 g/L NaCl, demonstrates osmotic resilience via lipid storage and delayed proliferation trade-offs.
2. Multi-omics reveal DNA replication upregulation and lipid catabolism suppression as keystones of chronic salt adaptation.
3. START2-mediated lipid droplet accumulation sustains membrane stability, validated by RNAi knockdown.
4. Controlled genomic instability emerges as an adaptive strategy for survival in hypersaline environments.
5. Mitochondrial-ER contact expansion prioritizes ATP synthesis.

## Data Availability

Data of all figures in this paper are available from the Supporting Information.

## SUPPLEMENTAL MATERIAL

Supplemental material msystems.01549-25-s0001.pdf Tables S1 to S7; Fig. S1 to S7.

ASM does not own the copyrights to Supplemental Material that may be linked to, or accessed through, an article. The authors have granted ASM a non-exclusive, world-wide license to publish the Supplemental Material files. Please contact the corresponding author directly for reuse.
