# Supplementary material for: Mitochondrial remodeling and metabolic reprogramming drive long-term salinity adaptation in Tetrahymena thermophila
Source: mSystems. 2025 Dec 23;11(2):e01549-25. doi: 10.1128/msystems.01549-25 (PMC12911363; doi:10.1128/msystems.01549-25)
Supplement: Supplemental material — Tables S1 to S7; Fig. S1 to S7. [file msystems.01549-25-s0001.pdf]

SupplementaryMaterials

Mitochondrial Remodeling and Metabolic Reprogramming Drive Long-Term Salinity Adaptation in *Tetrahymena thermophila*

Fengyu Yuan[1], Wenyu Li[1], Aiyun Li[1], Ting Tang[1], Yuming Zhang[1], Song Xie[1], Fengchao Li[1]*, Fengsong Liu[1, 2, 3]*

1 The Key Laboratory of Zoological Systematics and Application, College of Life Sciences, Hebei University, Baoding 071002, China

2 Hebei Basic Science Center for Biotic Interaction, Hebei University, Baoding 071002, China

3 Engineering Research Center of Ecological Safety and Conservation in Beijing-Tianjin-Hebei (Xiong'an New Area) of MOE, Baoding 071002, China.

* Corresponding author: liufengsong@hbu.edu.cn (LFS); lifengchao2000@126.com (LFC)

1. Method

1.1 Calculation of Growth Kinetic Parameters

Based on OD_{600} data from growth curves (Figure 1C), the maximum specific growth rate (μ) and generation time (Tg) were calculated for each strain. The exponential phase was first identified for each strain by selecting the time interval with the most rapid linear increase in ln(OD). The growth rate (μ) was then determined as the slope from linear regression of ln(OD) versus time during this phase (equation: ln(OD_t) = ln(OD_0) + μt). The final μ value for each strain is presented as the mean ± standard deviation of six biological replicates. Generation time (Tg) was subsequently calculated using the formula Tg = ln(2) / μ.

1.2. Metabolite Quantification and Enzymatic Activity Profiling

Reactive Oxygen Species (ROS): Intracellular ROS levels were quantified using 20,70-dichlorodihydrofluorescein diacetate (DCFH-DA) with fluorescence measured at 485/525 nm (Synergy HTX) [1], normalized to cell density (6 biological replicates).

Multi-metabolite Profiling: Malondialdehyde (MDA), glutathione (GSH), triacylglycerols (TAG), and activities of glutathione S-transferase (GST), total superoxide dismutase (T-SOD), and lipase (LPS) were determined via commercial kits. Biomarker levels were normalized to total protein (Bradford assay) across six replicates.

Calcium Flux Analysis: Cells (3×10^6) loaded with 2 μM Fura-2 AM (12°C, 40 min) were analyzed via dual-excitation ratiometry (λ_{ex}=340/380 nm, λ_{em}=510 nm) using fluorescence spectrophotometry (6 replicates).

Pyruvate Quantification: Cell lysates (8×10^6 cells) in 8% trichloroacetic acid were derivatized with 2,4-dinitrophenylhydrazine, with absorbance measured at 520 nm [2]. Data expressed as ng/10^6 cells.

Lactate HPLC Analysis: Sonicated cell extracts (4×10^6 cells) were separated on a Carbomix H-NP10 column (55°C, 0.8 mL/min 2.5 mM H_2SO_4), detecting lactate elution at 210 nm [3].

1.3. Mitochondrial Respirometry Analysis

Instrumentation & Sample Preparation: Mitochondrial complex activities (CI, CII, CIV) were analyzed using an Oxygraph-2k system. Cells ($2\text{-}4\times10^5$) were permeabilized with 5 μg digitonin in respiration buffer (110 mM sucrose, 20 mM HEPES, 10 mM KH_2PO_4, 0.5 mM EGTA, 3 mM $MgCl_2$, 60 mM K-lactate, 20 mM taurine, 0.1% BSA) at 30°C with 750 rpm stirring.

Substrate-Uncoupler-Inhibitor Titration (SUIT): 1. State 3/4 Respiration: CI activity was initiated with 5 mM glutamate + 4 mM malate, followed by 0.125 mM ADP (State 3). Respiratory control ratio (RCR) was calculated as State3/State4 after ADP depletion. **2. Complex-specific Profiling:** Sequential additions included 5 μM rotenone (CI inhibition), 10 mM succinate + 25 μM antimycin A (CII activation), and 2 mM ascorbate + 10 mM TMPD + 100 mM NaN_3 (CIV measurement). Cytochrome c (10 μM) confirmed membrane integrity [4, 5].

Intact Cell Metabolic Profiling: Basal respiration was established with 10 mM glucose, 1 mM pyruvate, and 2 mM glutamine. Substrate utilization was assessed via inhibitors: 25 μM etomoxir (FAO inhibition), 2 μM UK5099 (pyruvate transport), 3 μM BPTES (glutaminase). Mitochondrial coupling was evaluated through oligomycin (ATP synthase inhibition) and FCCP titration (uncoupling), followed by 10 μM rotenone/antimycin A [6].

Data Acquisition: DatLab software processed oxygen flux data, with six biological replicates per condition.

1.4. Transmission Electron Microscopy and Mitochondrial Morphometrics

T. thermophila cells were dual-fixed in 2.5% glutaraldehyde/2% osmium tetroxide (4°C, 10 min) followed by 1% osmium post-fixation (4°C, 1 h). Ethanol-dehydrated samples were embedded in TAAB 812 resin and sectioned (70-90 nm) using an ultramicrotome. Uranyl acetate/lead citrate double-stained sections were imaged at 80 kV (Hitachi HT7800 TEM).

For mitochondrial morphometric analysis, ≥50 cells per group were randomly selected. Mitochondrial cross-sectional areas were quantified from ≥20 organelles/cell using ImageJ via grayscale thresholding and contour tracing. Statistical significance was determined by one-way ANOVA across three biological replicates.

2. Tables And Figures

Table S1. The nucleotide sequences of the primers used in this study.

Primers name	Primers sequences（5’-3’）
RT-TtABC2-F	GCGGAAAGACTACCCTTGCT
RT-TtABC2-R	AGCTGCATGATCTGAGCCAA

RT-TtMFS-F	GTGAAGGTGCAACTCGCAAA
RT-TtMFS-R	AGAAATGGGAGAGTATGCCACA
RT-TtTP-F	TGGCTTACAGCGTGTTCCAT
RT-TtTP-R	GAAGCAGATAAAGCGCCTGC
RT-TtACAD-F	TCCCTTTGGGGAAGGAAGGA
RT-TtACAD-R	CTGTTAATGCCCAGCCTCCA
RT-TtACOX-F	TCGCATTTTACATCGATTGTTTGC
RT-TtACOX-R	GAGCTGAGAGCCTGCTTGAA
RT-TtGSTm46-F	ACAGAGGTCGTCCATAGCCT
RT-TtGSTm46-R	TGGAACACGTCTCTGTTAGA
RT-TtGSTm34-F	ACTGGGGCATCAGAGGTTTG
RT-TtGSTm34-R	TTGGGGAAGTCAAAGCCGAG
RT-TtPCNA-F	ATGGATTCAGCTCACGTTGC
RT-TtPCNA-R	GGTGTCGGGGATACCCAAAG
RT-TtLIG4-F	TGGCATAGGAGATTGGACAGA
RT-TtLIG4-R	ACCTGCTCCCACTTTAGCAA
RNAi-TtSTART2-F	CCCTCGAGATTTAGTAGCACTTGTTTATGAA
RNAi-TtSTART2-R	GGGGTACCATTTTGACTTTGTTCTTGAATCCTG
RNAi-TtKIN-F	CCCTCGAGTGGTCGTGAAAATCGTGCTG
RNAi-TtKIN-R	GGGGTACCTAAAGTCGGCAGGACCAACG
RNAi-GFP-F	CCCCTCGAGAATGGTGAGCAAGGGCGAGGA
RNAi-GFP-R	GGGGTACCACTTGTACAGCTCGTCCATGC
RT-TtSTART2-F	ATTCTTAGCATTCAGATTCG
RT-TtSTART2-R	TTTTTCACCTTTCTTCGG
17S-F	CCTGAGAAACGGCTACTACAACTA
17S-R	AAATGTTTACTCCCTAAGTCGAAC

Table S2. qRT-PCR standard-curve parameters

Gene	Slope	Efficiency (%)	y-intercept	R^2 (*)
ABC2	-3.352	98.8%	20.749	0.9948
MSF	-3.359	98.4%	19.770	0.9613
TP	-3.324	99.9%	21.222	0.9794
ACAD	-3.371	98.0%	23.220	0.9915

ACOX	-3.362	98.3%	23.244	0.9849
GSTm46	-3.367	98.1%	24.710	0.9897
GSTm34	-3.369	98.1%	25.310	0.9918
PCNA	-3.350	98.9%	20.636	0.9935
LIG4	-3.382	97.6%	21.881	0.9996
START2	-3.394	97.0%	22.537	0.9918
17S	-3.403	96.7%	15.154	0.9835

Table S3. Growth kinetic parameters of WT and ST (ST-4, ST-8, ST-12) strains during exponential phase.

Strain	Maximum Specific Growth Rate, μ (h^{-1})	Generation Time, Tg (h)
**WT**	0.046 ± 0.003	15.1 ± 1.0
**ST-4**	0.042 ± 0.002	16.5 ± 0.8
**ST-8**	0.038 ± 0.002	18.2 ± 1.0
**ST-12**	0.028 ± 0.001	24.8 ± 0.9

Values are presented as mean ± standard deviation (n=6).

Table S4. Sequencing and assembly statistics of the transcriptome data from different *T. thermophila* strains

sample	library	raw_reads	clean_reads	clean_bases	error_rate	Q30
WT-1	FRAS210184037-1r	47756934	46698776	7.0G	0.03	93.9
WT-2	FRAS210184038-1r	46807436	45482308	6.82G	0.02	94.27
WT-3	FRAS210184039-1r	43740192	42737330	6.41G	0.02	94.08
ST-8-1	FRAS210184043-1r	46480070	45443126	6.82G	0.03	93.54
ST-8-2	FRAS210184044-1r	43078866	41792914	6.27G	0.02	94.11
ST-8-3	FRAS210184045-1r	50186150	48837054	7.33G	0.02	94.25
ST-12-1	FRAS230098601-1r	41914212	40247296	6.04G	0.03	91.98
ST-12-2	FRAS230098602-1r	45596666	43709088	6.56G	0.03	92.26
ST-12-3	FRAS230098603-1r	43490784	41446560	6.22G	0.03	92.17

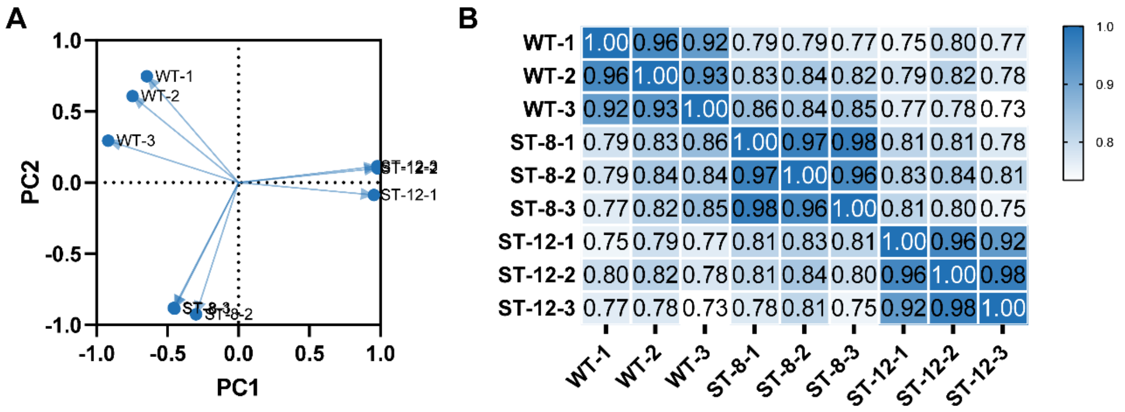

Figure S1. Quality assessment of all samples of the *T. thermophila* transcriptome. (A) Principal component analysis of all transcriptomic libraries. (B) Heatmap of Pearson correlation coefficient values. Correlation values close to 1 represent the high similarity between two samples.

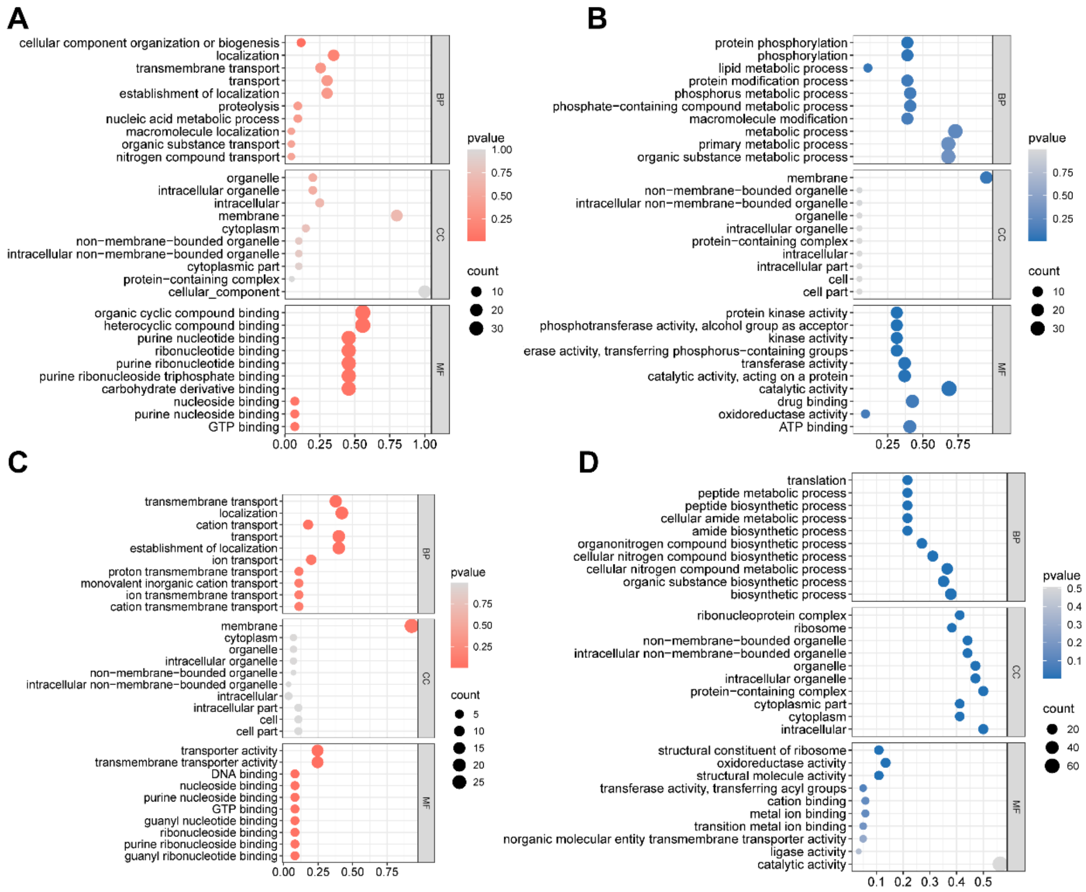

Figure S2. Systematic Gene Ontology (GO) enrichment profiling of differentially expressed genes (DEGs) in ST strains. A. GO term enrichment analysis for upregulated genes in ST-8 vs WT. B. Parallel analysis of downregulated genes in ST-8 vs WT. C.

Comparative GO landscape of upregulated genes in ST-12 vs WT. D. Downregulated gene ontology network in ST-12 vs WT.

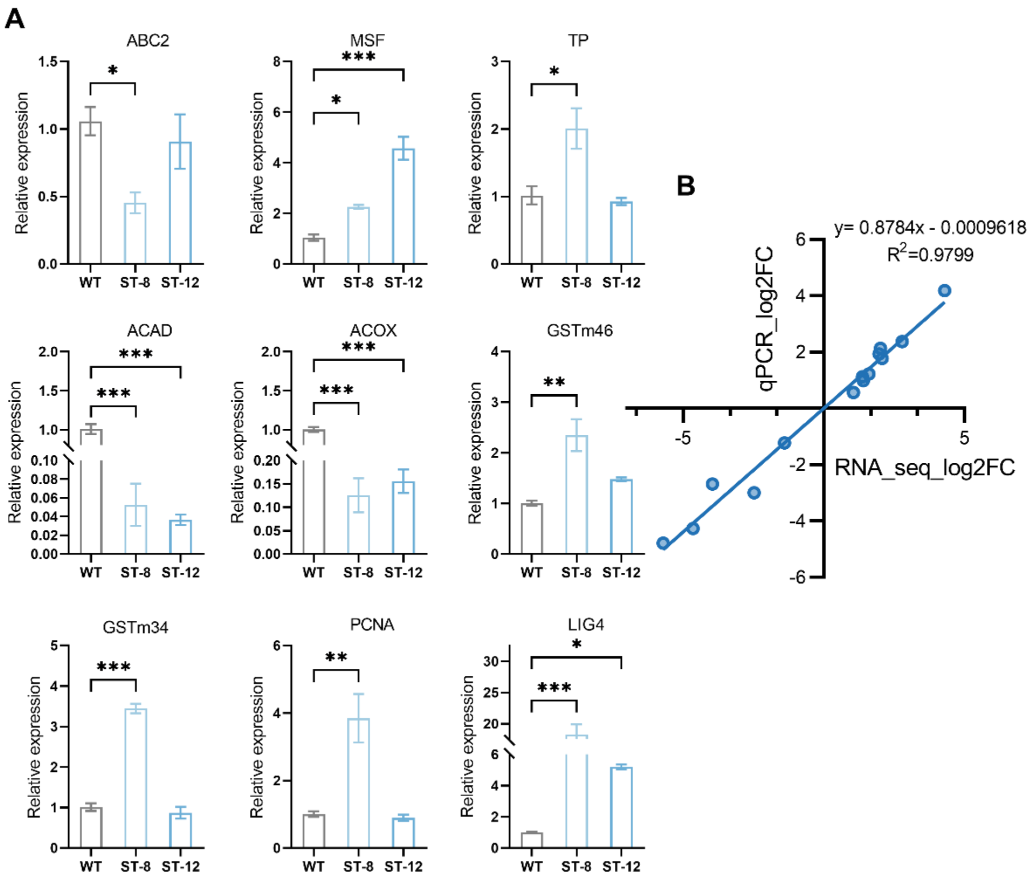

Figure S3. Relative expression levels of six DEGs withWT and ST-8 and ST-12. A. The nine genes expression as determined by qRT-PCR. B. Comparison between the log2 of gene expression ratios obtained from RNA-seq data and qRT-PCR. The qPCR log2 value of the expression ratio (ST-8/WT, ST-12/WT) (y-axis) was plotted against the value from the RNA-seq (x-axis). R^2 represented the correlation coefficient between qRT-PCR and RNA-seq.

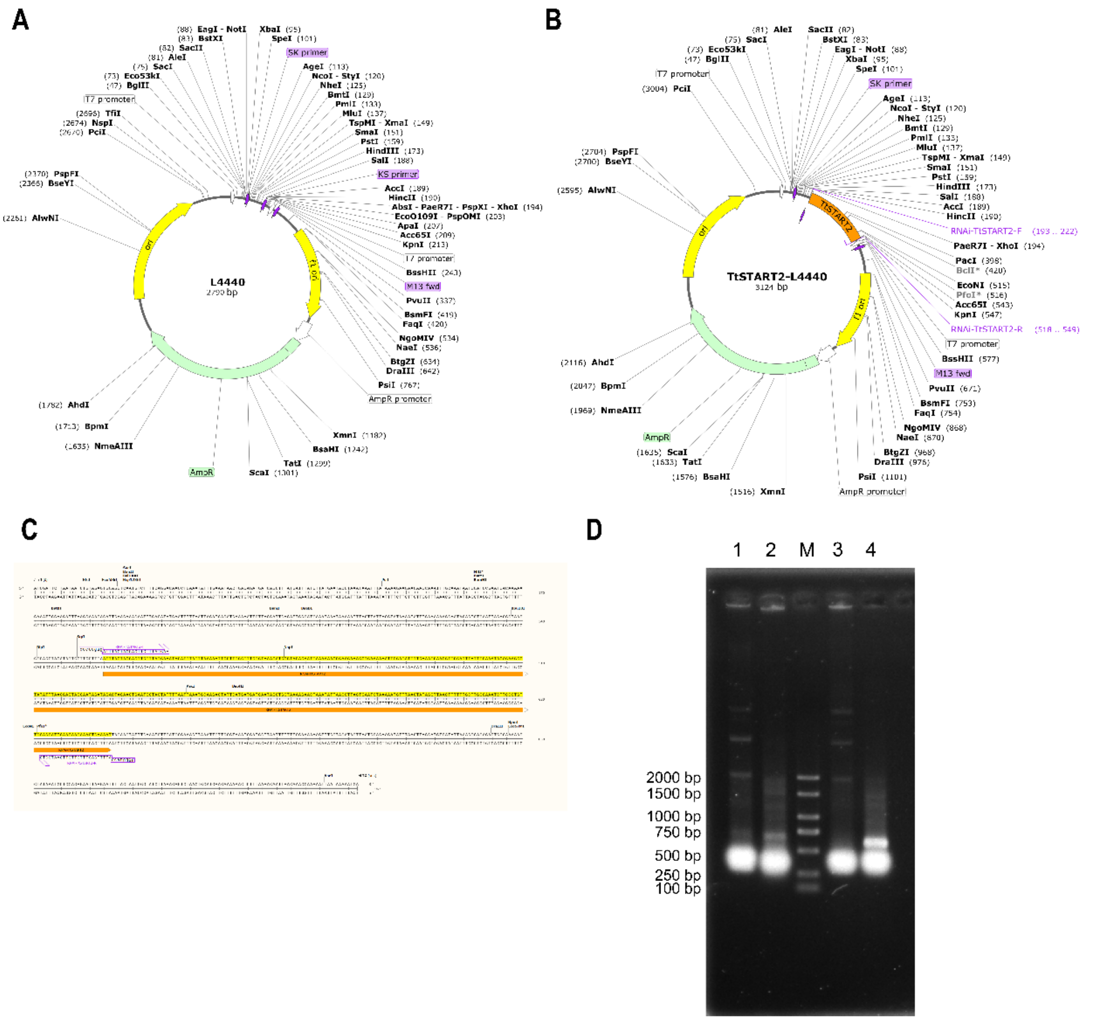

Figure S4. Development and verification of RNAi tools for TtSTART2 gene silencing. A-B. Schematic representations of the control (L4440) and experimental (RNAi-START2-L4440) vectors used for dsRNA expression. C. Illustration of the TtSTART2 gene sequence, with the specific RNAi target fragment indicated in orange. D. Validation of dsRNA production by IPTG induction. dsKIN (Lanes 1-2) and dsSTART2 (Lanes 3-4) were expressed in HT115(DE3) *E. coli* with (+) or without (-) induction. The results demonstrate successful and specific induction of the expected dsRNA fragments. M, DL2000 DNA Marker.

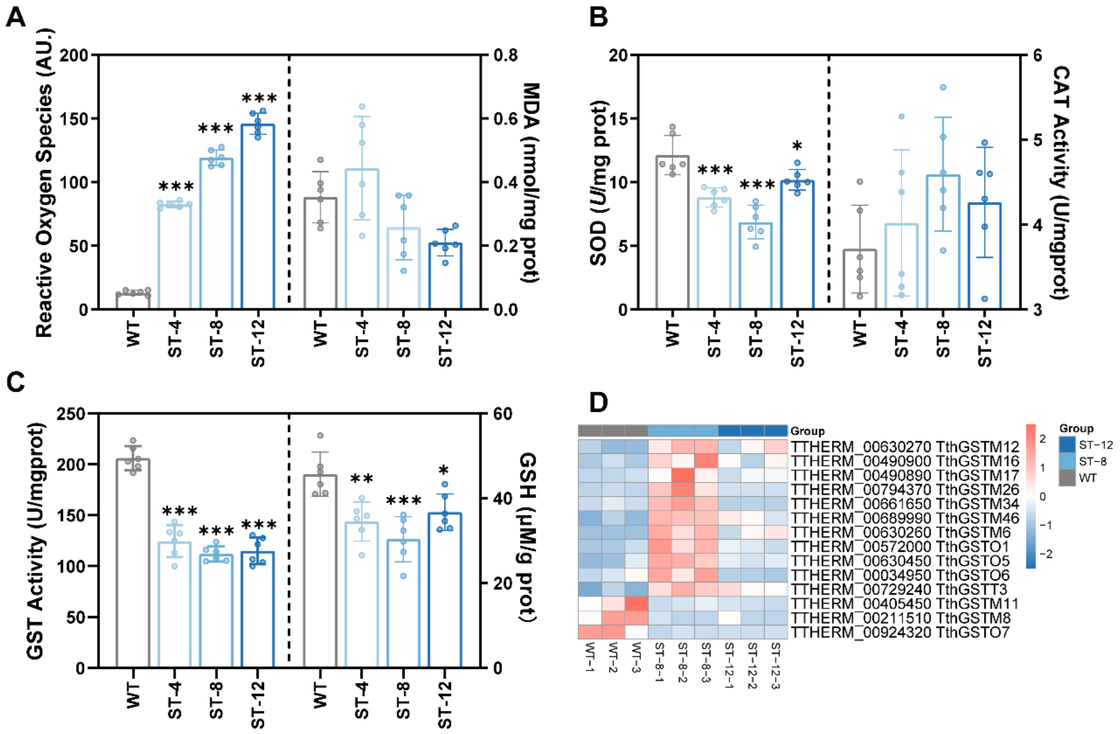

Figure S5. Systemic analysis of oxidative stress response and glutathione (GSH)-dependent detoxification pathways in ST strains. A. Quantification of reactive oxygen species (ROS) and malondialdehyde (MDA). (n = 6). Data normalized to total protein (nmol/mg). ***$p < 0.01$ vs. WT (unpaired t-test). B. Dynamic changes in antioxidant enzyme activities. Left: SOD activity. Right: CAT activity. C. Glutathione metabolism. Left: GST activity. Right: Total GSH. D. Genetic heat map of significant changes in the GST gene family in the ST-8 strain.

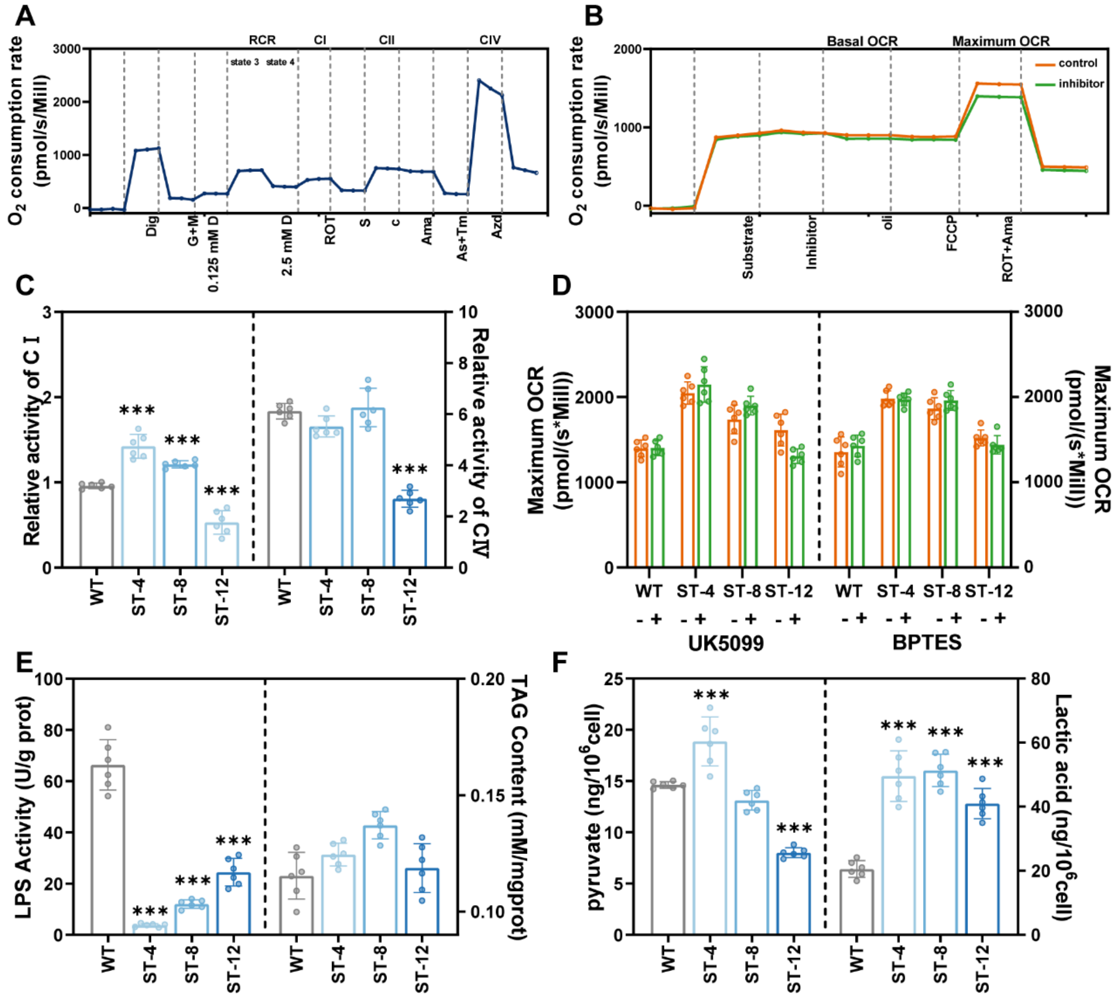

Figure S6. Mitochondrial metabolic plasticity and bioenergetic remodeling in ST strains. A. Experimental workflow for mitochondrial electron transport chain (ETC) complex activity assays. Key reagents: Digitonin (Dig, 0.015% w/v, membrane permeabilization), Malate (M, 5 mM), Glutamate (G, 5 mM), ADP-Mg^{2+} (D, 2.5 mM), Rotenone (Rot, 2 μM, complex I inhibitor), Succinate (S, 10 mM), Cytochrome c (c, 0.1 mM, complex IV substrate), Antimycin A (Ama, 1 μM, complex III inhibitor), Ascorbate (As, 10 mM)/TMPD (Tm, 0.5 mM, complex IV electron donors). Incubation: 28°C. Respiratory control ratio (RCR) quantification in ST strains. RCR = State 3 (ADP-stimulated)/State 4 (basal) respiration measured by Clark electrode (Oxygraph-2k, Oroboros). Data from 6 biological replicates. B. Substrate utilization preference assay. Experimental design: Cells incubated with substrates-long-chain fatty acids (LCFA, palmitate-BSA), glucose, pyruvate, glutamine ± metabolic inhibitors: Etomoxir, UK5099, BPTES. Respiratory parameters

measured by O2K after sequential injection of oligomycin, rotenone/antimycin A. C. Relative activities of mitochondrial complexes I and IV under substrate-limited conditions. D. Time-resolved oxygen consumption rate (OCR) dynamics post-inhibition. E. Metabolite quantification. Left: Lipopolysaccharide (LPS) levels by limulus amebocyte lysate assay. Right: Triacylglycerol (TAG) content via enzymatic colorimetry. F. Pyruvate and lactate homeostasis. ***$p < 0.001$.

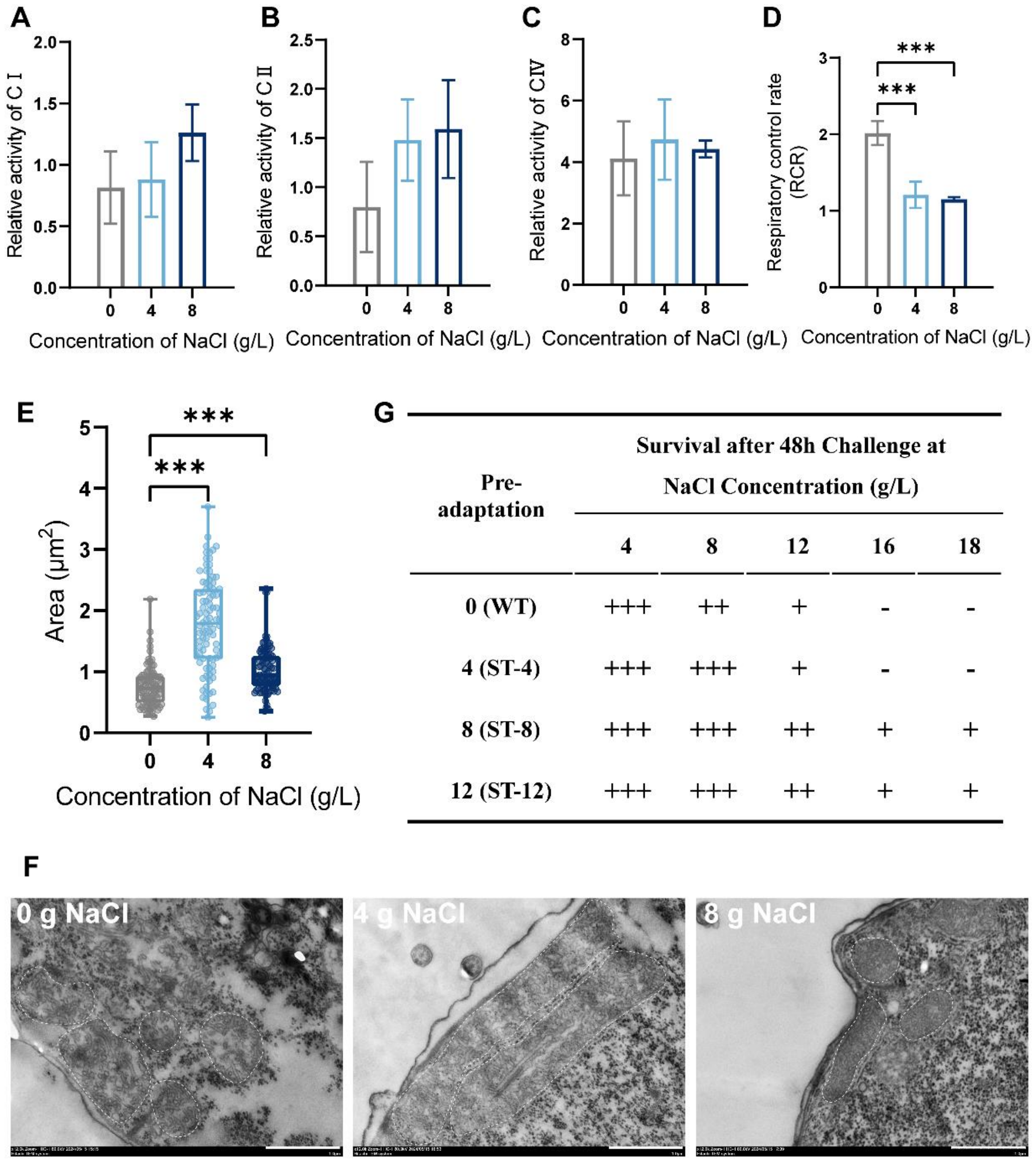

Pre-adaptation	Survival after 48h Challenge at NaCl Concentration (g/L)				
	4	8	12	16	18
0 (WT)	+++	++	+	-	-
4 (ST-4)	+++	+++	+	-	-
8 (ST-8)	+++	+++	++	+	+
12 (ST-12)	+++	+++	++	+	+

Figure S7. Characterization of acute stress in wild-type (WT) and validation of long-term adaptation in salt-tolerant (ST) strains. A-D. Mitochondrial respiratory function in WT cells under acute sublethal salt stress (24 h exposure to 4 and 8 g/L NaCl). A-

C. Activities of mitochondrial complexes I (CI, A), II (CII, B), and IV (CIV, C) were measured. D. Respiratory control ratio (RCR) was calculated to assess oxidative phosphorylation coupling efficiency. E. Quantification of mitochondrial cross-sectional area from TEM images in WT cells under the indicated conditions ($n \geq 95$ cells per group from three independent experiments). F. Representative transmission electron microscopy (TEM) images showing mitochondrial morphology in WT cells under acute salt stress. Scale bar, 1 μm. G. Survival of WT and ST strains challenged with lethal concentrations of NaCl (12 to 18 g/L) for 48 hours. Survival and growth were visually assessed and scored as: +++, robust growth; ++, inhibited growth but surviving; +, viable but no growth; -, non-viable.

Table S5. The first 500 genes up-regulated in the ST-8 strain. The remaining 260 genes, excluding hypothetical proteins.

gene_id	log2FoldChange (ST-8)	log2FoldChange (ST-12)	gene_name	gene_description
**immobilization antigen**				
g16681	19.25	16.92	TTHERM_00594350	SerL immobilization antigen
g16680	18.28	16.18	TTHERM_00594360	immobilization antigen
g16679	16.52	14.74	TTHERM_00594370	immobilization antigen
g24931	10.44	-3.43	TTHERM_00363070	immobilization antigen
g28148	7.38	3.77	TTHERM_00739534	Giardia Variant Surface Antigen
g16675	6.97	8.13	TTHERM_00595440	immobilization antigen
g24930	6.90	-0.14	TTHERM_00363060	immobilization antigen
g24929	6.73	0.45	TTHERM_00363050	immobilization antigen
g16943	6.72	7.39	TTHERM_01178710	Giardia Variant Surface Antigen
g24927	6.50	0.62	TTHERM_00363030	immobilization antigen
g17945	6.08	#N/A	TTHERM_00819480	Cell surface immobilisation antigen SerH
g21215	6.03	-0.38	TTHERM_01101580	Cell surface immobilisation antigen SerH
g23388	5.61	5.01	TTHERM_00854200	Cell surface immobilisation antigen SerH
g18013	5.20	2.54	TTHERM_01026170	Cell surface immobilisation antigen SerH
g24918	5.01	0.71	TTHERM_00362970	immobilization antigen
g24928	4.81	-0.10	TTHERM_00363040	immobilization antigen
**leishmanolysin family protein**				
g19427	13.39	12.32	TTHERM_00112760	leishmanolysin family protein
g27944	10.16	7.16	TTHERM_00541549	leishmanolysin family protein
g734	8.22	7.07	TTHERM_00541550	leishmanolysin family protein
g20950	5.76	8.01	TTHERM_01082860	leishmanolysin family protein
g729	5.19	2.92	TTHERM_00543570	leishmanolysin family protein
g11532	4.44	1.29	TTHERM_00289550	leishmanolysin family protein
**transmembrane protein**				

g20771	12.86	14.54	TTHERM_00621540	transmembrane protein putative
g24136	12.76	9.37	TTHERM_00423260	transmembrane protein putative
g11937	9.53	12.01	TTHERM_00418160	transmembrane protein putative
g22562	9.20	9.80	TTHERM_00957680	transmembrane protein putative
g1248	8.05	8.40	TTHERM_01197000	transmembrane protein putative
g11978	7.51	2.81	TTHERM_00415800	transmembrane protein putative
g6002	6.92	3.67	TTHERM_01104820	transmembrane protein putative
g10689	6.83	0.55	TTHERM_01159960	transmembrane protein putative
g13139	6.80	1.55	TTHERM_00220910	transmembrane protein putative
g10175	6.36	7.88	TTHERM_00327040	transmembrane protein putative
g17802	6.04	6.54	TTHERM_00201730	transmembrane protein putative
g19499	5.94	2.84	TTHERM_00574320	transmembrane protein putative
g16547	5.90	3.38	TTHERM_00582400	transmembrane protein putative
g3773	5.85	2.84	TTHERM_01433610	transmembrane protein putative
g8322	5.74	6.18	TTHERM_00419840	transmembrane protein putative
g22563	5.64	6.16	TTHERM_00957670	transmembrane protein putative
g25051	5.48	#N/A	TTHERM_00809320	transmembrane protein putative
g454	5.36	5.40	TTHERM_00497980	transmembrane protein putative
g23151	5.36	2.14	TTHERM_00994370	transmembrane protein putative
g16480	5.27	6.19	TTHERM_00581730	transmembrane protein putative
g22565	5.01	3.19	TTHERM_00957650	transmembrane protein putative
g728	4.66	2.15	TTHERM_00543580	transmembrane protein putative
g7576	4.49	3.86	TTHERM_00465000	transmembrane protein putative
g2510	4.40	8.43	TTHERM_00823840	transmembrane protein putative
g13907	4.37	2.91	TTHERM_00188620	transmembrane protein putative
g20046	4.31	3.62	TTHERM_00092870	transmembrane protein putative
g22173	4.14	3.01	TTHERM_00490810	transmembrane protein putative
g11858	3.91	4.48	TTHERM_00277320	transmembrane protein putative
g23373	3.90	-0.09	TTHERM_00233120	transmembrane protein putative
g23366	3.84	-2.18	TTHERM_00233000	transmembrane protein putative

**tetraspanin family protein**				
g2417	9.69	5.36	TTHERM_00865200	tetraspanin family protein
g2415	4.59	-1.52	TTHERM_00865180	tetraspanin family protein
g2416	4.42	-3.54	TTHERM_00865190	tetraspanin family protein
**nucleotide-sugar transporter**				
g25689	9.13	7.53	TTHERM_00011160	nucleotide-sugar transporter
**transmembrane amino acid transporter protein**				
g22962	8.99	13.73	TTHERM_01188330	transmembrane amino acid transporter protein
g22963	8.06	10.72	TTHERM_01188340	transmembrane amino acid transporter protein
**Trk system potassium uptake protein TrkA**				
g16507	8.93	5.91	TTHERM_00581980	Trk system potassium uptake protein TrkA amine-terminal domain protein
g16512	7.96	3.67	TTHERM_00582030	Trk system potassium uptake protein TrkA amine-terminal domain protein
g16501	6.77	6.29	TTHERM_00581930	Trk system potassium uptake protein TrkA amine-terminal domain protein
g16504	6.09	2.92	TTHERM_00581959	Trk system potassium uptake protein TrkA amine-terminal domain protein
g16513	6.05	2.42	TTHERM_00582039	Trk system potassium uptake protein TrkA amine-terminal domain protein
g16510	5.71	0.22	TTHERM_00582010	Trk system potassium uptake protein TrkA amine-terminal domain protein
g16511	4.71	0.90	TTHERM_00582020	Trk system potassium uptake protein TrkA amine-terminal domain protein
g16509	4.19	0.40	TTHERM_00582000	Trk system potassium uptake protein TrkA amine-terminal domain protein
**nematoblast specific protein**				
g7134	8.88	10.66	TTHERM_00721890	nematoblast specific protein
g7135	4.74	6.86	TTHERM_00721900	nematoblast specific protein
**ABC transporter**				

g21188	8.70	4.43	TTHERM_01417320	ABC transporter family protein
g15861	4.44	4.77	TTHERM_00550900	ABC-type multidrug transport system ATPase and permease component
g21720	4.09	3.93	TTHERM_00034920	ABC transporter family protein
**kinase domain protein**				
g27294	8.58	4.87	TTHERM_01068945	TKL Ser/Thr protein kinase
g18330	8.54	7.31	TTHERM_00058410	Serine/Threonine kinase domain protein
g8270	7.16	5.14	TTHERM_00455370	Serine/Threonine kinase domain protein
g27359	7.12	4.17	TTHERM_01044740	kinase domain protein
g372	5.72	1.43	TTHERM_00497250	Serine/Threonine kinase domain protein
g25063	5.47	1.40	TTHERM_00809420	kinase domain protein
g20462	5.17	4.05	TTHERM_00355710	kinase domain protein
g25172	5.11	0.86	TTHERM_01125110	Tyrosine-protein kinase ephrin type A/B receptor-like
g16945	4.96	7.11	TTHERM_01178750	TKL Ser/Thr protein kinase
g2652	4.41	1.42	TTHERM_00825440	TOP2 DNA topoisomerase IV
g14033	4.29	5.91	TTHERM_00190800	choline/ethanolamine kinase
g74	4.21	2.67	TTHERM_01194670	Serine/Threonine kinase domain protein
g29230	4.08	3.99	TTHERM_01358412	kinase domain protein
g25992	4.04	3.35	TTHERM_00388260	kinase domain protein
g19007	3.91	1.69	TTHERM_00048990	inorganic polyphosphate/ATP-NAD kinase
**tetratricopeptide repeat protein (TPR)**				
g11892	8.33	7.02	TTHERM_00418620	tetratricopeptide repeat protein
g27472	7.78	6.12	TTHERM_00308825	Tetratricopeptide-like helical domain superfamily
g29479	7.21	7.62	TTHERM_00125775	Tetratricopeptide-like helical domain superfamily
g25584	5.38	2.78	TTHERM_00013180	tetratricopeptide repeat protein
g18552	4.86	4.11	TTHERM_00274579	Tetratricopeptide-like helical domain superfamily

**P1/s1 nuclease**				
g5360	7.84	1.55	TTHERM_00559800	P1/s1 nuclease
g5359	4.67	2.08	TTHERM_00559790	P1/s1 nuclease
**proteinase inhibitor**				
g5741	7.74	0.07	TTHERM_00627070	kazal-type proteinase inhibitor 1
g5740	7.62	-0.29	TTHERM_00627080	kazal-type proteinase inhibitor 1
g5746	5.26	-1.21	TTHERM_00627030	kazal-type proteinase inhibitor 1
g3214	4.15	3.11	TTHERM_00691540	proteinase inhibitor I4 serpin
g5744	3.99	-0.63	TTHERM_00627050	kazal-type proteinase inhibitor 1
g22330	3.98	1.97	TTHERM_00954270	proteinase inhibitor I4 serpin
**phage head-tail family protein**				
g21369	7.65	0.39	TTHERM_00735390	phage head-tail family protein putative
**lipase**				
g5170	7.65	6.72	TTHERM_00624220	lipase family protein
**ATPase**				
g5794	7.61	6.92	TTHERM_00923160	ATPase P-type (transporting) HAD superfamily protein
g18197	5.55	9.92	TTHERM_00061570	Na-H/K antiporter P-type ATPase alpha subunit family protein
g2399	4.55	3.18	TTHERM_00865050	ORC1 origin recognition complex subunit 1 putative
g5795	4.11	4.06	TTHERM_00923150	E1-E2 ATPase family protein
g12793	3.92	4.77	TTHERM_00322860	P-type family IC HAD ATPase
**papain family cysteine protease**				
g16182	7.60	6.47	TTHERM_00079630	papain family cysteine protease
g4243	7.30	1.65	TTHERM_00660420	papain family cysteine protease
g4248	4.33	2.30	TTHERM_00660390	papain family cysteine protease
g6574	3.92	10.84	TTHERM_00525070	papain family cysteine protease
**alpha/beta fold hydrolase**				
g8468	7.50	6.01	TTHERM_00476480	alpha/beta fold hydrolase

g21935	5.61	2.92	TTHERM_00654080	alpha/beta fold hydrolase
g16886	4.36	3.73	TTHERM_00433870	alpha/beta fold hydrolase
**kelch motif protein**				
g5376	7.46	6.08	TTHERM_00560030	kelch motif protein
g2135	5.30	4.48	TTHERM_00969590	kelch motif protein
**oxidoreductase**				
g13733	7.44	5.38	TTHERM_00145050	zinc-binding dehydrogenase family oxidoreductase
g11106	6.10	6.08	TTHERM_00317340	zinc-binding dehydrogenase family oxidoreductase
g5992	5.77	5.87	TTHERM_00666170	NmrA-like oxidoreductase
g13730	5.73	7.06	TTHERM_00145070	zinc-binding dehydrogenase family oxidoreductase
g19994	5.09	0.90	TTHERM_00187260	oxidoreductase short chain dehydrogenase/reductase family protein
g3713	4.83	2.65	TTHERM_00694470	FAD/FMN-binding family oxidoreductase
g18513	4.79	4.98	TTHERM_00160800	FAD/FMN-binding family oxidoreductase
g14462	4.69	4.06	TTHERM_00151670	zinc-binding dehydrogenase family oxidoreductase
g13731	4.26	2.11	TTHERM_00145060	zinc-binding dehydrogenase family oxidoreductase
g11040	4.17	3.73	TTHERM_00298310	aldo/keto reductase family oxidoreductase
g14425	4.04	1.58	TTHERM_00152000	oxidoreductase short chain dehydrogenase/reductase family protein
**Leucine-rich repeat domain superfamily**				
g4246	7.30	9.42	TTHERM_00660400	Leucine-rich repeat domain superfamily
g26581	5.45	6.27	TTHERM_00442243	Leucine-rich repeat domain superfamily
g7097	4.73	2.09	TTHERM_00377300	Leucine-rich repeat domain superfamily
**Protein of unknown function DUF4441**				
g25583	6.99	2.60	TTHERM_00013190	Protein of unknown function DUF4441

g21085	5.47	-0.85	TTHERM_00036860	Protein of unknown function DUF4441
**kinesin**				
g15099	6.87	6.78	TTHERM_00115410	Kinesin-like protein
g14409	4.68	2.61	TTHERM_00152170	kinesin motor catalytic domain protein
**GTPase**				
g24400	6.81	8.91	TTHERM_00382270	ADP-ribosylation factor(Arf)/Arf-like (Arl) small GTPase family protein
g17757	5.65	3.48	TTHERM_00207230	Ras family small GTPase
g15149	5.12	3.98	TTHERM_00129450	P-loop containing nucleoside triphosphate hydrolase
**heme/steroid-binding domain protein**				
g19093	6.79	3.42	TTHERM_00051880	cytochrome b5-like heme/steroid-binding domain protein
g4145	3.94	6.64	TTHERM_00689870	Cytochrome b5-like heme/steroid binding domain
**zinc finger protein**				
g11900	6.76	6.02	TTHERM_00418560	U1 zinc finger protein
g28789	5.92	7.84	TTHERM_00008730	Zinc finger
g27801	5.52	5.35	TTHERM_00112784	B-box-type zinc finger
g17106	5.42	1.96	TTHERM_01323740	zinc finger protein
g21390	5.20	8.46	TTHERM_01367690	zinc finger LSD1 subclass family protein
g16261	4.77	2.46	TTHERM_00077830	Zinc finger, ZZ-type superfamily
g9036	4.77	3.68	TTHERM_00412150	Zinc finger, RING/FYVE/PHD-type
g15475	4.45	2.17	TTHERM_00131330	AN1-type zinc finger protein
g13906	4.44	0.31	TTHERM_00188610	Zinc finger, RanBP2-type superfamily
g18449	4.33	1.13	TTHERM_00161480	CHY zinc finger protein
g10515	4.25	6.56	TTHERM_00310690	RING-H2 zinc finger protein
g4955	3.98	4.76	TTHERM_00600240	Zinc finger, RING/FYVE/PHD-type
**Growth factor receptor cysteine-rich domain superfamily**				

g24048	6.72	5.16	TTHERM_00100060	Growth factor receptor cysteine-rich domain superfamily
g10045	6.10	6.58	TTHERM_00942820	Growth factor receptor cysteine-rich domain superfamily
g23537	4.11	5.24	TTHERM_01364620	Growth factor receptor cysteine-rich domain superfamily
g11461	3.96	5.99	TTHERM_00292190	Growth factor receptor cysteine-rich domain superfamily
**Forkhead-associated (FHA)**				
g20114	6.64	5.08	TTHERM_00089230	Forkhead-associated (FHA) domain
**antioxidant enzyme**				
g16992	6.64	5.62	TTHERM_00630260	GST6 glutathione S-transferase amine-terminal domain protein
g1893	5.33	4.06	TTHERM_01084110	glutamine synthetase
g22164	4.85	2.93	TTHERM_00490890	GST17 glutathione S-transferase amine-terminal domain protein
g22163	4.11	2.66	TTHERM_00490900	GST16 glutathione S-transferase amine-terminal domain protein
g18816	3.89	3.56	TTHERM_00046090	GPX8 phospholipid hydroperoxide glutathione peroxidase
g16993	3.84	3.16	TTHERM_00630270	GST12 glutathione S-transferase amine-terminal domain protein
**MFS transporter**				
g21040	6.28	2.03	TTHERM_00798160	oxalate/formate antiporter family transporter
g23233	5.65	6.27	TTHERM_00355794	MFS transporter superfamily
g1013	5.33	2.42	TTHERM_01207680	oxalate/formate exchange protein
g8093	5.29	3.50	TTHERM_00437690	MFS transporter
g108	4.08	2.33	TTHERM_00684670	MFS transporter
**acetyltransferase**				
g10433	6.16	3.77	TTHERM_00335770	arylamine N-acetyltransferase

**integral membrane protein**				
g20568	6.12	7.32	TTHERM_00876960	integral membrane protein DUF6 containing protein
g20570	4.79	0.03	TTHERM_00876930	integral membrane protein DUF6 containing protein
g20569	4.74	4.35	TTHERM_00876950	integral membrane protein DUF6 containing protein
g19770	4.07	-2.51	TTHERM_00169120	YeeE/YedE family protein
g9268	3.95	1.59	TTHERM_00401850	integral membrane protein DUF6 containing protein
**methionine aminopeptidase**				
g5188	5.99	6.24	TTHERM_00624400	type II methionine aminopeptidase
**OsmC**				
g9172	5.79	2.79	TTHERM_00408840	OsmC family protein
**bowman-birk serine protease inhibitor (BBI)**				
g21810	5.71	0.18	TTHERM_00030140	bowman-birk serine protease inhibitor family protein
g5830	5.27	2.31	TTHERM_00791970	bowman-birk serine protease inhibitor family protein
**von willebrand factor type A (VWA)**				
g14841	4.85	5.26	TTHERM_01478580	von willebrand factor type A domain protein
g5399	4.82	4.72	TTHERM_00561250	von willebrand factor type A (VWA) domain was originally protein
g3268	4.44	4.31	TTHERM_00760220	von willebrand factor type A domain protein
**histidine phosphatase**				
g11994	5.70	4.69	TTHERM_00415620	histidine phosphatase family (branch 2) protein
**Retinoblastoma protein (RB)**				
g8061	5.47	2.54	TTHERM_00439030	retinoblastoma-associated-like protein
**thioester hydrolase**				
g9571	5.25	4.75	TTHERM_00371200	acyl-CoA thioester hydrolase

**endo-1-4-beta-xylanase**				
g11571	5.09	3.38	TTHERM_00289180	endo-1-4-beta-xylanase xylA putative
g25227	3.90	1.75	TTHERM_01050420	endo-1-4-beta-xylanase xylA putative
**heat shock 70 kDa protein**				
g1436	5.18	-2.04	TTHERM_01080440	SSA4 heat shock 70 kDa protein
g23940	4.20	-0.07	TTHERM_00105110	HSP70 heat shock 70 kDa protein
g14985	3.90	-4.17	TTHERM_00125640	SSA3 heat shock 70 kDa protein
**cyclic nucleotide-binding domain protein**				
g19261	4.77	0.54	TTHERM_00122480	cyclic nucleotide-binding domain protein
g315	4.14	3.35	TTHERM_00470560	cyclic nucleotide-binding domain protein
g25025	3.92	0.75	TTHERM_00808080	cyclic nucleotide-binding domain protein
**Rab-GAP-TBC domain superfamily**				
g29223	4.67	3.63	TTHERM_01041980	Rab-GAP-TBC domain superfamily
g24591	4.45	4.04	TTHERM_01041940	Rab-GAP-TBC domain superfamily
**other protein**				
g18268	6.67	-0.74	TTHERM_00058950	AhpD-like
g22104	6.35	3.78	TTHERM_01008770	Polymorphic outer membrane protein repeat
g16621	6.23	4.21	TTHERM_00718030	chromosome condensation regulator RCC1 repeat protein
g13147	6.22	4.81	TTHERM_00220820	LMBR1-like motif protein
g12429	5.93	3.28	TTHERM_00391470	Phospholipase C, phosphatidylinositol-specific, Y domain;Phospholipase C, phosphatidylinositol-specific, Y domain
g8297	5.83	0.99	TTHERM_00455120	Nucleotide exchange factor Fes1
g10988	5.80	3.25	TTHERM_00299879	WAG1 hypothetical protein
g4180	5.74	8.30	TTHERM_00663990	NAD(P)-dependent epimerase/dehydratase-related protein
g28891	5.64	6.73	TTHERM_00544138	Cysteine and histidine-rich domain-containing protein 1
g11970	5.63	4.80	TTHERM_00417840	H-type lectin domain protein

g24289	5.59	7.08	TTHERM_00377240	Dbl homology (DH) domain
g13993	5.52	#N/A	TTHERM_00189440	SDS22/Internalin LRR-containing
g2825	5.47	6.38	TTHERM_00723610	UDH10 UvrD/REP family helicase
g4546	5.47	5.33	TTHERM_00659050	ImpB/MucB/SamB family protein
g4304	5.42	2.97	TTHERM_00616010	dienelactone hydrolase family protein
g2287	5.28	3.45	TTHERM_00925490	GPCR6 G protein coupled glucose receptor regulating Gpa2 protein
g14770	5.18	-0.53	TTHERM_01088000	REJ domain protein
g9014	5.16	5.35	TTHERM_00487160	P-loop containing nucleoside triphosphate hydrolase
g9713	4.98	6.99	TTHERM_00519803	Myo-inositol-1-phosphate synthase
g15696	4.92	6.10	TTHERM_00313800	kynurenine-oxoglutarate transaminase
g23204	4.87	3.77	TTHERM_00224550	H-type lectin domain protein
g28899	4.82	1.89	TTHERM_00991025	Leucine-rich repeat
g23945	4.81	5.03	TTHERM_00105060	DRH11 DEAD/DEAH-box helicase family protein
g27683	4.79	5.18	TTHERM_00623769	Centrosomal CEP44 domain
g25991	4.77	4.22	TTHERM_00388250	SNF2 family amine-terminal protein
g12654	4.77	3.31	TTHERM_01156850	class I glutamine amidotransferase
g13232	4.77	2.11	TTHERM_00219050	MXR1 peptide methionine sulfoxide reductase msrA
g17305	4.63	7.63	TTHERM_00264940	Gtr1/RagA G motif protein
g5223	4.62	0.55	TTHERM_00624740	Rdx family protein
g28078	4.61	1.67	TTHERM_00452046	SKP1/BTB/POZ domain superfamily
g5808	4.56	2.91	TTHERM_00923010	STAC1/2/3
g3770	4.54	1.46	TTHERM_01433580	CHN4 class I chitinase
g14766	4.51	4.58	TTHERM_01087940	UbiE/COQ5 family methyltransferase
g17371	4.50	5.12	TTHERM_00637360	Gar1/NAF1 RNA-binding region protein
g20727	4.48	3.11	TTHERM_00691008	EamA domain
g3880	4.48	2.83	TTHERM_00773520	XPG I-region protein

g12321	4.45	3.40	TTHERM_00394700	WD40-repeat-containing domain superfamily
g5725	4.44	3.52	TTHERM_00627230	Myb-like DNA-binding domain protein
g25717	4.40	1.96	TTHERM_00008790	WAMTP1-MAML2 fusion protein putative
g18634	4.35	0.83	TTHERM_00155590	HMG box protein putative
g24549	4.33	5.02	TTHERM_00463835	Pectinacetylesterase/NOTUM
g25972	4.30	2.78	TTHERM_00387050	LIG4 ATP-dependent DNA ligase
g7351	4.27	5.43	TTHERM_00529980	josephin protein
g18122	4.23	1.80	TTHERM_00069250	targeting protein for Xklp2 protein
g22477	4.21	3.13	TTHERM_00143770	inorganic pyrophosphatase
g24435	4.21	3.56	TTHERM_00383640	cullin protein neddylation domain protein
g20060	4.21	1.48	TTHERM_00091780	ankyrin repeat protein putative
g20225	4.19	3.81	TTHERM_00085310	enoyl-(acyl carrier) reductase
g18754	4.16	2.21	TTHERM_00644650	START domain protein
g8615	4.13	1.92	TTHERM_00473110	ARS1 alanine-tRNA ligase putative
g16336	4.12	3.60	TTHERM_00077120	pyrimidine 5-prime-nucleotidase
g11252	4.07	0.93	TTHERM_00297160	ESP1 peptidase family C50 protein
g12042	4.06	3.62	TTHERM_00927988	Sulphide quinone-reductase
g21458	4.06	2.46	TTHERM_00042640	exocyst complex component Sec6
g22500	4.02	3.44	TTHERM_00860470	AMP-binding enzyme family protein
g20654	4.01	3.04	TTHERM_00945250	ALV1 hypothetical protein
g9400	4.01	3.86	TTHERM_00448920	serine carboxypeptidase family protein
g1520	4.00	1.45	TTHERM_01205340	IP3 receptor calcium ion channel protein
g4488	4.00	1.51	TTHERM_00657530	NIF system FeS cluster assembly, NifU, N-terminal
g164	3.99	3.62	TTHERM_00686170	solute carrier family protein
g13708	3.99	3.13	TTHERM_00145310	Man1-Src1p-carboxy-terminal domain protein
g28414	3.97	3.71	TTHERM_00752775	Lysosomal cystine transporter
g5192	3.97	0.58	TTHERM_00624450	CYC20 amine-terminal domain cyclin
g27901	3.97	3.14	TTHERM_00488194	XMAP215/Dis1/CLASP, TOG domain

g20971	3.92	3.51	TTHERM_00989450	Aromatic amino acid beta-eliminating lyase/threonine aldolase
g16869	3.89	4.49	TTHERM_00433700	4-hydroxybenzoate synthetase (chorismate lyase) protein putative
g26552	3.89	1.65	TTHERM_00094115	RNA recognition motif domain
g2246	3.87	1.60	TTHERM_00850580	EF hand protein

Table S6. The first 500 genes down-regulated in the ST-8 strain. The remaining 358 genes, excluding hypothetical proteins.

gene_id	log2FoldChange (ST-8)	log2FoldChange (ST-12)	gene_name	gene_description
**glycerophosphodiester phosphodiesterase**				
g16803	-11.59	-6.91	TTHERM_00430070	glycerophosphodiester phosphodiesterase
**Domain of unknown function (DUF)**				
g15033	-11.46	-3.06	TTHERM_00127190	Protein of unknown function DUF4441
g10312	-7.93	-0.52	TTHERM_00340100	Protein of unknown function DUF4441
g6003	-7.40	-2.30	TTHERM_01104840	Protein of unknown function DUF4441
g9248	-7.19	-0.70	TTHERM_00399690	Protein of unknown function DUF4441
g5994	-7.14	-2.34	TTHERM_00666130	Protein of unknown function DUF4441
g20098	-7.13	-4.31	TTHERM_00090430	Protein of unknown function DUF4441
g15077	-6.71	0.23	TTHERM_00128590	Protein of unknown function DUF4441
g1008	-6.67	-2.68	TTHERM_01207730	Protein of unknown function DUF4441
g20976	-6.15	-0.81	TTHERM_00770862	Protein of unknown function DUF4441
g6004	-6.11	-7.49	TTHERM_01104850	Protein of unknown function DUF4441
g21779	-5.91	-0.63	TTHERM_00399580	Protein of unknown function DUF4441
g29012	-5.84	-2.53	TTHERM_00013195	Protein of unknown function DUF4441
g14339	-5.33	-4.03	TTHERM_00635780	Protein of unknown function DUF676, lipase-like
g20001	-5.15	-4.28	TTHERM_00094320	Protein of unknown function DUF4441

g24195	-5.08	-3.42	TTHERM_01309160	Protein of unknown function DUF924
g23009	-4.59	-2.10	TTHERM_00770830	Protein of unknown function DUF4441
g15452	-4.53	-0.48	TTHERM_00133530	Protein of unknown function DUF4441
g16818	-4.43	-0.56	TTHERM_00431190	Protein of unknown function DUF4441
g28013	-4.16	-2.91	TTHERM_00180990	Protein of unknown function DUF4441
g24335	-3.85	-0.81	TTHERM_00378730	Protein of unknown function DUF4441
g7143	-3.84	-6.77	TTHERM_00997470	Protein of unknown function DUF3472
g19493	-3.70	-2.54	TTHERM_00574260	Protein of unknown function DUF5077
g20032	-3.65	-2.21	TTHERM_00094020	Protein of unknown function DUF4441
**DCD1A/B-like**				
g11107	-11.38	-9.92	TTHERM_00317330	acid ceramidase-like protein putative
**MFS transporter**				
g236	-10.55	-10.80	TTHERM_00471390	CAT14 MFS transporter
g1619	-8.77	-7.58	TTHERM_01055440	Major facilitator superfamily domain-containing protein 1
g25984	-7.98	-8.82	TTHERM_00388160	MFS transporter
g11086	-6.02	-5.05	TTHERM_00318540	MFS transporter, Myo-inositol Transporter
g21025	-5.87	-0.91	TTHERM_01100520	Major Facilitator Superfamily Riboflavin Transporter
g13823	-5.58	1.67	TTHERM_02091560	MFS transporter putative
g2319	-4.45	-3.54	TTHERM_00787330	Major Facilitator Superfamily Sugar Transporters
g9045	-3.82	-0.37	TTHERM_00412050	MFS transporter, Myo-inositol Transporter
g19379	-3.70	-4.69	TTHERM_00113260	Major facilitator superfamily
g11625	-3.68	-4.46	TTHERM_00285570	Major facilitator, sugar transporter-like
**Acyl-CoA oxidase**				
g16169	-10.14	-4.96	TTHERM_00079730	Acyl-CoA oxidase
g13893	-4.64	-5.70	TTHERM_00188450	Acyl-coenzyme A oxidase 4-like
g10785	-4.42	-2.81	TTHERM_00463290	Acyl-CoA oxidase
**Amino acid transporter, transmembrane domain**				

g15318	-9.93	-9.30	TTHERM_00137700	Amino acid transporter, transmembrane domain
g5232	-5.62	0.71	TTHERM_00624820	Amino acid transporter, transmembrane domain
**Alpha/Beta hydrolase fold**				
g12510	-8.94	-8.54	TTHERM_00784250	Alpha/Beta hydrolase fold
g12861	-8.71	-8.19	TTHERM_00320120	Secreted Mono/Diacylglycerol Lipase
g7027	-5.26	-0.54	TTHERM_00537110	Alpha/Beta hydrolase fold
g20593	-4.47	-0.49	TTHERM_00927200	Peptidase S10, serine carboxypeptidase
g6776	-4.41	-3.22	TTHERM_00516360	Alpha/beta hydrolase fold-1
g19410	-4.20	-2.70	TTHERM_00112930	TGL3 ab-hydrolase associated lipase region protein; Partial AB-hydrolase lipase domain
g20594	-4.19	0.71	TTHERM_00927190	Peptidase S10, serine carboxypeptidase
g17513	-4.16	-4.00	TTHERM_00193970	Fungal lipase-like domain
g7030	-4.02	3.24	TTHERM_00537150	Esterase, PHB depolymerase
g20019	-3.79	-2.64	TTHERM_00094160	Potential DNA-binding regulatory protein
g17572	-3.73	-2.45	TTHERM_00194590	Fungal lipase-like domain
g27084	-3.73	-0.54	TTHERM_00732575	Monoacylglycerol and Diacylglycerol Lipase
g16088	-3.64	-1.85	TTHERM_00859240	Alpha/Beta hydrolase fold
**dioxygenase**				
g21661	-8.92	-8.47	TTHERM_00035520	Indoleamine 2,3-dioxygenase
g2275	-5.08	-2.81	TTHERM_00925630	Phytanoyl-CoA dioxygenase-like
g24893	-3.63	-3.57	TTHERM_00361730	Mitochondrial Persulfide Dioxygenase
**kinase domain protein**				
g25882	-8.52	-2.23	TTHERM_00000030	kinase domain protein
g9838	-7.59	-1.68	TTHERM_00343300	Serine/Threonine kinase domain protein
g21394	-7.42	-2.41	TTHERM_00900600	kinase domain protein
g5396	-7.24	-4.37	TTHERM_00561220	Calcium-dependent Serine/Threonine Protein Kinases
g26211	-7.16	-1.48	TTHERM_00257010	kinase domain protein

g16356	-6.72	-1.05	TTHERM_00075900	kinase domain protein
g21287	-5.54	-1.35	TTHERM_01640980	kinase domain protein
g4976	-5.29	-1.41	TTHERM_00600470	kinase domain protein
g22370	-5.06	-2.42	TTHERM_01377900	kinase domain protein
g24744	-4.91	-1.97	TTHERM_00751030	kinase domain protein
g21335	-4.90	-2.80	TTHERM_00734050	Threonine-specific kinase-like protein
g16471	-4.69	-8.90	TTHERM_00581640	A-kinase anchor protein 7 isoform gamma
g19381	-4.60	-1.58	TTHERM_00113249	Leucine-rich repeat
g21101	-4.60	-0.66	TTHERM_01406900	kinase domain protein
g281	-4.40	-1.87	TTHERM_00470900	kinase domain protein
g16917	-4.32	0.94	TTHERM_01090230	Threonine-specific kinase-like protein
g5141	-4.30	-2.98	TTHERM_00622950	Calcium-dependent Serine/Threonine Protein Kinases
g12088	-4.12	-2.49	TTHERM_00886980	kinase domain protein
g12260	-4.01	-1.75	TTHERM_00266410	L-seryl-tRNA(Sec) kinase
g15851	-3.98	-0.82	TTHERM_00550830	Two-component system histidine kinase
g7036	-3.97	-0.54	TTHERM_00537200	Two-component system histidine kinase
g20949	-3.97	0.16	TTHERM_01082870	Serine/Threonine Kinases and Pseudokinases
g16143	-3.91	-0.87	TTHERM_00080000	Mitogen-activated protein kinase
g26604	-3.81	-2.47	TTHERM_00764585	cAMP-dependent kinase regulatory chain
g6915	-3.81	-1.59	TTHERM_01956349	kinase domain protein
g22553	-3.79	-1.66	TTHERM_00958790	Serine/threonine-protein kinase Atg1-like
g22368	-3.69	-1.70	TTHERM_01376880	kinase domain protein
g12827	-3.60	-2.04	TTHERM_00320520	Two-component system histidine kinase
g15304	-3.58	-1.06	TTHERM_01002810	Two-component system histidine kinase
**phosphatase family**				
g11986	-8.51	-6.12	TTHERM_00415690	Ser/thr phosphatase family protein
g3209	-6.97	-7.29	TTHERM_00691490	Ser/thr phosphatase family protein
g8131	-4.70	0.19	TTHERM_00437400	Ser/thr phosphatase family protein

**aminotransferase**				
g4229	-8.23	-5.59	TTHERM_00661540	Kynurenine--oxoglutarate transaminase
g19775	-4.95	-3.15	TTHERM_00170170	Aminotransferase class V domain
**Growth factor receptor cysteine-rich domain superfamily**				
g28292	-8.03	-9.37	TTHERM_00554976	Growth factor receptor cysteine-rich domain superfamily
g2270	-7.59	-1.89	TTHERM_00925710	PKD/REJ-like domain
g23602	-6.86	-1.77	TTHERM_01144960	R-spondin domain-containing protein
g19361	-6.20	-1.17	TTHERM_00115430	Growth factor receptor cysteine-rich domain superfamily
g25512	-6.12	-0.21	TTHERM_00014950	Growth factor receptor domain 4
g9109	-6.00	-1.61	TTHERM_00411410	Growth factor receptor cysteine-rich domain superfamily
g16495	-5.69	-0.09	TTHERM_00581870	Growth factor receptor cysteine-rich domain superfamily
g19362	-5.61	5.68	TTHERM_00115420	Growth factor receptor domain 4
g11954	-5.47	1.34	TTHERM_00417960	H-type lectin domain
g24167	-5.43	-2.50	TTHERM_00424530	Growth factor receptor cysteine-rich domain superfamily
g27760	-5.34	0.00	TTHERM_00415819	H-type lectin domain
g27284	-5.10	-2.37	TTHERM_00555313	Growth factor receptor cysteine-rich domain superfamily
g10046	-4.91	-3.85	TTHERM_00942810	Growth factor receptor cysteine-rich domain superfamily
g14056	-4.69	-1.86	TTHERM_00647200	H-type lectin domain
g27219	-4.47	-2.18	TTHERM_00756047	Quinoprotein alcohol dehydrogenase-like superfamily
g27007	-4.42	-3.76	TTHERM_00096781	Growth factor receptor cysteine-rich domain superfamily

g24161	-4.40	-1.80	TTHERM_00424470	Growth factor receptor cysteine-rich domain superfamily
g20220	-4.37	0.22	TTHERM_00085340	Growth factor receptor cysteine-rich domain superfamily
g27214	-4.24	-5.08	TTHERM_00417975	R-spondin domain-containing protein
g252	-4.19	-2.31	TTHERM_01230168	Pectin lyase fold/virulence factor
g22400	-4.08	-4.19	TTHERM_01393290	PKD/REJ-like domain
g25197	-4.00	-1.76	TTHERM_00885680	R-spondin, Fu-CRD domain
g12160	-3.86	-0.06	TTHERM_00268340	Growth factor receptor cysteine-rich domain superfamily
g20670	-3.73	-0.61	TTHERM_00944150	Growth factor receptor cysteine-rich domain superfamily
g14893	-3.68	3.36	TTHERM_01321560	PKD/REJ-like domain
g21559	-3.59	-5.24	TTHERM_00037610	Growth factor receptor cysteine-rich domain superfamily
g5261	-3.56	-3.66	TTHERM_00578570	Quinoprotein alcohol dehydrogenase-like superfamily
**AMP-binding enzyme**				
g24585	-7.97	0.39	TTHERM_01042000	AMP-binding enzyme family protein
g15909	-7.59	-6.52	TTHERM_00554420	AMP-dependent synthetase/ligase domain
g19991	-6.31	-3.91	TTHERM_00187230	AMP-dependent synthetase/ligase domain
g19891	-6.22	-1.10	TTHERM_00185130	AMP-binding enzyme family protein
g22509	-5.79	-0.99	TTHERM_00860420	AMP-binding enzyme family protein
g23101	-5.24	-3.62	TTHERM_00790570	AMP-dependent synthetase/ligase domain
g5393	-3.68	-3.59	TTHERM_00561190	AMP-dependent synthetase/ligase domain
**Potassium channel, calcium-activated, SK**				
g8416	-7.79	-5.11	TTHERM_00420820	Potassium channel, calcium-activated, SK
**zinc finger**				
g18677	-7.76	-3.57	TTHERM_00638880	zinc finger lsd1 subclass family protein;
g28008	-6.26	-7.62	TTHERM_00648945	Zinc finger, RING/FYVE/PHD-type

g5011	-6.14	-0.51	TTHERM_00600830	Zinc finger, UBR-type
g24867	-5.44	0.01	TTHERM_00361420	Zinc-finger domain of monoamine-oxidase A repressor R1
g15065	-5.09	1.50	TTHERM_00128480	Zinc finger C2H2-type
g13082	-4.85	2.37	TTHERM_00655530	Zinc finger C2H3-type
g8092	-4.04	-1.90	TTHERM_00437700	zinc finger transcription factor sma protein putative
g16095	-3.71	-1.26	TTHERM_00858110	Zinc finger, C3HC4 RING-type
g19443	-3.65	0.80	TTHERM_00112590	RING-H2 finger domain-containing protein
g2185	-3.63	-1.48	TTHERM_01002770	Zinc finger C2H2-type
**LITAF-like zinc ribbon domain**				
g20919	-7.60	-7.19	TTHERM_01068060	LITAF domain containing protein
g24638	-5.81	-1.77	TTHERM_01138240	LPS-induced tumour necrosis factor alpha factor
**immobilization antigen**				
g24164	-7.48	-4.28	TTHERM_01700340	immobilization antigen
g12723	-7.10	-0.74	TTHERM_00775860	immobilization antigen
g28744	-6.09	-4.03	TTHERM_00424474	Cell surface immobilisation antigen SerH
g22717	-6.00	-6.98	TTHERM_00606960	Cell surface immobilisation antigen SerH
g22208	-5.97	-0.74	TTHERM_00489510	Cell surface immobilisation antigen SerH
g22714	-4.37	-3.54	TTHERM_00606950	Cell surface immobilisation antigen SerH
g21327	-4.18	-2.92	TTHERM_00733950	Cell surface immobilisation antigen SerH
**Ran GTPase-activating protein**				
g952	-7.41	0.86	TTHERM_01260710	Ran GTPase-activating protein
g6310	-6.60	-1.38	TTHERM_00612584	Ran GTPase-activating protein
g26950	-6.54	-2.32	TTHERM_01442295	Ran GTPase-activating protein
g29286	-6.47	-2.32	TTHERM_01292740	Ran GTPase-activating protein
g16533	-6.35	-3.88	TTHERM_00582240	Ran GTPase-activating protein
g21145	-5.46	0.80	TTHERM_00874780	Ran GTPase-activating protein
g26677	-5.30	1.18	TTHERM_02164828	Ran GTPase-activating protein

g16206	-4.47	-1.77	TTHERM_00079420	Ran GTPase-activating protein
g16500	-4.29	-0.92	TTHERM_00581920	Ran GTPase-activating protein
g28749	-4.20	-0.60	TTHERM_00748152	Ran GTPase-activating protein
g1202	-4.16	-1.11	TTHERM_01151460	Ran GTPase-activating protein
g21658	-4.04	-0.95	TTHERM_01651032	RAN GTPase-activating protein 1/2
g27492	-3.98	0.26	TTHERM_00324350	Ran GTPase-activating protein
g10868	-3.98	-0.92	TTHERM_00630736	Ran GTPase-activating protein
g24973	-3.94	-0.32	TTHERM_01441770	Ran GTPase-activating protein
g13554	-3.84	-1.28	TTHERM_00242220	RAN GTPase-activating protein 1/2
g19126	-3.76	-0.83	TTHERM_00052150	Ran GTPase-activating protein
**pectinacetylesterase family protein**				
g12839	-7.28	-1.84	TTHERM_00320350	Pectinacetylesterase/NOTUM
g17967	-5.86	-4.31	TTHERM_00820720	Pectinacetylesterase/NOTUM
**Ferric reductase**				
g6755	-7.20	-3.59	TTHERM_00513180	Respiratory burst oxidase/Ferric reductase
g6760	-5.04	-2.03	TTHERM_00515210	Respiratory burst oxidase/Ferric reductase
**MORN repeat**				
g22941	-6.83	-2.99	TTHERM_01225357	MORN repeat
**papain family cysteine protease**				
g16357	-6.83	-7.57	TTHERM_00075890	CTH65 papain family cysteine protease; Peptidase C1A
g13098	-6.34	-1.73	TTHERM_00655340	Papain-like cysteine peptidase superfamily
g16186	-6.32	-7.39	TTHERM_00079600	CTH109 papain family cysteine protease; Peptidase C1A
g17078	-5.62	-6.97	TTHERM_00895720	CTH83 papain family cysteine protease; Peptidase C1A
g7972	-4.35	-5.70	TTHERM_00532060	CTH98 papain family cysteine protease; Peptidase C1A
g25302	-4.27	-7.46	TTHERM_01354270	CTH106 papain family cysteine protease; Peptidase C1A

g23974	-4.26	-2.80	TTHERM_00102770	CTH36 papain family cysteine protease
**Potassium/sodium hyperpolarization-activated cyclic nucleotide-gated channel**				
g13719	-6.81	-2.05	TTHERM_00145180	Potassium/sodium hyperpolarization-activated cyclic nucleotide-gated channel
g17252	-6.71	-2.08	TTHERM_00263410	Voltage-gated potassium channel family H
g249	-6.00	-1.88	TTHERM_00471270	Cyclic nucleotide-binding domain-containing protein 1/2
g14376	-5.82	-1.73	TTHERM_00961900	Cyclic nucleotide-binding domain-containing protein 1/3
g16401	-5.72	-3.22	TTHERM_00075460	Potassium/sodium hyperpolarization-activated cyclic nucleotide-gated channel
g8546	-5.60	-0.75	TTHERM_00481240	Potassium/sodium hyperpolarization-activated cyclic nucleotide-gated channel
g3917	-5.45	1.70	TTHERM_00680610	Potassium/sodium hyperpolarization-activated cyclic nucleotide-gated channel
g25039	-5.32	-0.91	TTHERM_00809210	Potassium/sodium hyperpolarization-activated cyclic nucleotide-gated channel
g3667	-4.75	-0.10	TTHERM_00697170	Potassium/sodium hyperpolarization-activated cyclic nucleotide-gated channel
g4855	-4.49	0.29	TTHERM_00759050	Potassium/sodium hyperpolarization-activated cyclic nucleotide-gated channel
g10159	-4.35	-2.78	TTHERM_00326880	Cyclic nucleotide-binding domain superfamily
g1933	-4.33	-1.72	TTHERM_00998880	Cyclic nucleotide-binding domain
g7958	-4.20	-2.88	TTHERM_00532200	Cyclic nucleotide-binding domain-containing protein 1/2
g19568	-4.11	-2.52	TTHERM_00577070	Cyclic nucleotide-binding domain-containing protein 1/2
g1011	-3.99	-2.04	TTHERM_01207700	Cyclic nucleotide-binding domain-containing protein 1/2

g16748	-3.91	-3.71	TTHERM_01338570	Voltage-gated potassium channel family H
g17248	-3.87	-0.41	TTHERM_00263390	Potassium/sodium hyperpolarization-activated cyclic nucleotide-gated channel
g11622	-3.80	-1.49	TTHERM_00285600	Cyclic nucleotide-binding domain superfamily
g1400	-3.76	-1.23	TTHERM_01107440	Cyclic nucleotide-binding domain-containing protein 1/2
**transmembrane protein**				
g12788	-6.81	-0.10	TTHERM_00322890	Quinoprotein amine dehydrogenase, beta chain-like
g24794	-6.65	0.66	TTHERM_00753580	transmembrane protein putative
g20003	-6.32	-0.55	TTHERM_00094290	transmembrane protein putative
g19804	-6.30	-1.39	TTHERM_00170450	transmembrane protein putative;LVIVD
g17292	-6.13	-3.24	TTHERM_00264810	Tiny macrocysts signaling regulators
g20783	-5.90	-7.27	TTHERM_01012060	transmembrane protein putative
g11223	-5.88	0.12	TTHERM_00316110	transmembrane protein putative
g23758	-5.84	-1.66	TTHERM_00245810	transmembrane protein putative
g25115	-5.81	-1.89	TTHERM_00974070	Polymorphic outer membrane protein repeat
g21167	-5.78	-1.05	TTHERM_00872600	transmembrane protein putative
g26446	-5.66	-9.27	TTHERM_01009880	transmembrane protein putative
g25316	-5.64	0.53	TTHERM_00976520	Cadherin-like superfamily
g18334	-5.53	-3.04	TTHERM_00058360	transmembrane protein putative
g12009	-5.47	0.96	TTHERM_00414490	transmembrane protein putative
g22545	-5.45	-10.34	TTHERM_00935460	transmembrane protein putative
g25828	-5.42	-1.53	TTHERM_00002630	transmembrane protein putative
g20598	-5.39	-3.74	TTHERM_00927130	transmembrane protein putative
g25388	-5.28	-0.65	TTHERM_00024110	transmembrane protein putative
g17202	-5.27	-1.16	TTHERM_00261920	transmembrane protein putative
g16229	-5.27	-0.76	TTHERM_00079150	GCR1-cAMP receptor
g2865	-5.16	-7.56	TTHERM_00732700	Archaemetzincin

g11525	-5.11	-6.79	TTHERM_00753670	transmembrane protein putative
g12439	-5.06	-2.26	TTHERM_00391360	transmembrane protein putative
g5389	-4.92	1.21	TTHERM_00561150	Aspartic peptidase domain superfamily
g6703	-4.84	-3.57	TTHERM_00503730	transmembrane protein putative
g24942	-4.82	-0.81	TTHERM_00363160	Pectin lyase fold/virulence factor
g9613	-4.77	-1.22	TTHERM_00372660	transmembrane protein putative
g5281	-4.63	-1.60	TTHERM_00578760	transmembrane protein putative
g19805	-4.62	-3.03	TTHERM_00170460	transmembrane protein putative;LVIVD
g286	-4.46	-3.02	TTHERM_00470850	Tiny macrocysts signaling regulators
g4675	-4.45	0.34	TTHERM_00670620	transmembrane protein putative
g12923	-4.29	-0.33	TTHERM_01150340	transmembrane protein putative
g26240	-4.24	-0.98	TTHERM_00255700	transmembrane protein putative
g9603	-4.23	-2.83	TTHERM_00372540	transmembrane protein putative
g18191	-4.21	-1.91	TTHERM_00061610	Tiny macrocysts signaling regulators
g17874	-4.13	-1.25	TTHERM_00197990	transmembrane protein putative
g19959	-3.99	-1.56	TTHERM_00185860	Bacteriophage lysozyme-like
g19954	-3.74	-2.34	TTHERM_00185790	transmembrane protein putative
g2216	-3.73	0.54	TTHERM_00849290	transmembrane protein putative
g12109	-3.70	0.61	TTHERM_00275780	transmembrane protein putative
g22110	-3.70	-0.31	TTHERM_01008720	transmembrane protein putative
g15994	-3.70	-3.05	TTHERM_00846940	transmembrane protein putative
g10914	-3.68	-1.99	TTHERM_00300560	Tiny macrocysts signaling regulators
g14989	-3.67	-1.32	TTHERM_00125700	transmembrane protein putative
g9912	-3.66	2.58	TTHERM_00344200	transmembrane protein putative
g6283	-3.62	-3.57	TTHERM_00502720	transmembrane protein putative
g21948	-3.59	-0.61	TTHERM_00654220	transmembrane protein putative
g20065	-3.59	-3.14	TTHERM_00091730	transmembrane protein putative
**von Willebrand factor A-like domain**				
g8091	-6.64	-3.67	TTHERM_00437740	Uncharacterised protein family CoxE-like

g24271	-4.58	-1.84	TTHERM_00427560	von Willebrand factor A-like domain superfamily
g21705	-3.58	2.55	TTHERM_00035110	Calcium-activated chloride channel regulator
**ABC transporter-like, ATP-binding domain**				
g21680	-6.60	-0.82	TTHERM_00035350	ATP-binding cassette subfamily G transporters
g24150	-6.29	-5.50	TTHERM_00423380	Type 1 protein exporter
g9144	-5.94	-3.26	TTHERM_00409090	ATP-binding cassette subfamily G transporters
g12869	-5.70	-2.73	TTHERM_00320020	ATP-binding cassette transporter C
g28660	-4.79	-1.71	TTHERM_00693545	ABC transporter A
g22472	-4.73	-4.62	TTHERM_00862740	ABC transporter A
g7904	-4.68	-2.56	TTHERM_00532790	ABC transporter A
g21682	-4.66	-3.39	TTHERM_00035330	ATP-binding cassette subfamily G transporters
g20288	-4.28	-0.74	TTHERM_00083790	Type 1 protein exporter
g29667	-3.90	-1.93	TTHERM_00693800	ABC transporter A
**Histidine phosphatase superfamily (branch 2)**				
g26797	-6.52	-3.94	TTHERM_00056055	Histidine Acid Phosphatase
**Nucleoside transporter 11**				
g21338	-6.52	-2.05	TTHERM_00734080	NTP11 equilibrative nucleoside transporter family protein
**Pectin lyase fold/virulence factor**				
g7333	-6.40	-1.63	TTHERM_00757530	Pectin lyase fold/virulence factor
g10574	-4.14	-3.68	TTHERM_00310060	Pectin lyase fold/virulence factor
g17133	-3.81	-2.35	TTHERM_01405860	Pectin lyase fold/virulence factor
**Mitochondrial carrier protein**				
g10729	-6.37	-6.59	TTHERM_02139630	MCF8 ADP/ATP carrier protein
**Leucine-rich repeat domain superfamily**				
g12310	-6.27	-0.72	TTHERM_01598644	BspA-type LRR region

g28720	-6.03	-3.28	TTHERM_01865411	Leucine-rich repeat
g27224	-5.90	-1.68	TTHERM_01303791	NOD-like receptor (NLR)
g26590	-5.60	-0.76	TTHERM_01165330	Leucine-rich repeat
g9811	-5.33	-1.92	TTHERM_01605678	Leucine-rich repeat
g27977	-5.30	1.11	TTHERM_01147390	Dynein regulatory complex subunit 5
g28972	-5.30	-0.09	TTHERM_01121095	Leucine-rich repeat domain superfamily
g19241	-5.26	-1.36	TTHERM_00123640	Leucine-rich repeat
g26526	-5.03	-2.37	TTHERM_01478582	Leucine-rich repeat
g28761	-4.86	-3.87	TTHERM_00188685	Leucine-rich repeat domain superfamily
g23953	-4.34	-0.81	TTHERM_00862784	Dynein regulatory complex subunit 5
g24429	-4.25	-2.56	TTHERM_00935445	Leucine-rich repeat domain superfamily
g27618	-4.18	-3.92	TTHERM_01305186	Leucine-rich repeat
g27489	-4.17	-0.28	TTHERM_01577294	Leucine-rich repeat
g1492	-3.98	-0.86	TTHERM_01381116	Leucine-rich repeat
g15007	-3.87	-0.55	TTHERM_01445960	Leucine-rich repeat
g28582	-3.79	-1.94	TTHERM_00086815	Leucine-rich repeat domain superfamily
g27726	-3.77	-1.74	TTHERM_01160475	Leucine-rich repeat
g21131	-3.64	-2.02	TTHERM_01532654	Leucine-rich repeat
**tetratricopeptide repeat protein**				
g15087	-6.14	-0.79	TTHERM_00128700	Tetratricopeptide repeat
g3777	-5.55	-2.44	TTHERM_00147690	Periplasmic sensor-like domain superfamily
g14858	-5.52	-8.73	TTHERM_01359450	Tetratricopeptide repeat
g27364	-4.90	0.13	TTHERM_00263270	Tetratricopeptide-like helical domain superfamily
g15010	-4.39	-0.91	TTHERM_00126920	Tetratricopeptide repeat
g11429	-3.99	-0.40	TTHERM_00294500	Tetratricopeptide repeat
g1683	-3.89	-2.64	TTHERM_01079160	Tetratricopeptide repeat
g4131	-3.60	-2.67	TTHERM_00688750	Tetratricopeptide-like helical domain superfamily
**FAD dependent oxidoreductase**				

g4926	-5.92	-5.56	TTHERM_00599910	FAD dependent oxidoreductase
g1279	-3.91	-2.46	TTHERM_01093610	FAD dependent oxidoreductase
**Ion transport domain**				
g24437	-5.89	0.86	TTHERM_00300069	Ion transport domain
g15061	-3.73	-0.82	TTHERM_00128440	Ion transport domain
**nuclease**				
g8673	-5.87	-4.37	TTHERM_00474740	5'-deoxynucleotidase YfbR/HDDC2
g22621	-5.86	-2.35	TTHERM_01046820	GTPase GIMA/IAN/Toc
g23178	-5.60	-2.88	TTHERM_00222300	Ribonuclease T2-like
g22623	-5.49	-3.04	TTHERM_01045780	GTPase GIMA/IAN/Toc
g6015	-5.29	-3.13	TTHERM_01104960	Beta-Like Tubulin 1, BLT1
g22622	-5.28	-0.99	TTHERM_01046810	GTPase GIMA/IAN/Toc
g9467	-5.24	-2.11	TTHERM_00449740	P-loop containing nucleoside triphosphate hydrolase
g17290	-5.21	-1.79	TTHERM_00264790	Ribonuclease T2-like
g4193	-4.27	-3.43	TTHERM_00663830	XRN3 XRN 5-prime-3-prime exonuclease amine-terminal protein
g26696	-4.12	-3.36	TTHERM_00815160	Ribonuclease T2-like
g27209	-3.91	-2.04	TTHERM_00653655	Exonuclease MUT-7-like
**Ricin-type beta-trefoil lectin domain-like**				
g29630	-5.86	-5.37	TTHERM_00492404	Ricin B, lectin domain
**WD40-repeat-containing domain superfamily**				
g19134	-5.78	-2.11	TTHERM_00052210	WD40-repeat-containing domain superfamily
g12102	-5.55	-2.50	TTHERM_00275860	WD40-repeat-containing domain superfamily
g22193	-4.11	-2.90	TTHERM_00490660	WD40-repeat-containing domain superfamily
g3807	-4.10	-0.79	TTHERM_00827100	WD40-repeat-containing domain superfamily
g14128	-4.03	-2.39	TTHERM_00191740	WD40-repeat-containing domain superfamily
**P-type ATPase**				
g6729	-5.24	-3.19	TTHERM_00508970	TPA5 Na-H/K antiporter P-type ATPase alpha subunit family protein

g28600	-4.12	-0.06	TTHERM_00532407	Voltage-gated potassium channel family H
g8424	-4.05	-5.07	TTHERM_00420900	Cation transport ATPase (P-type)
g25337	-3.72	-2.07	TTHERM_00977720	FLP15 phospholipid-translocating P-type ATPase flippase family protein
**Protein phosphatase**				
g1665	-5.58	-2.77	TTHERM_01016230	Protein-tyrosine phosphatase-like
g26220	-5.58	-5.53	TTHERM_00256930	Protein-tyrosine phosphatase-like
g24316	-5.17	0.60	TTHERM_00378510	Dual specificity phosphatase, catalytic domain
g11199	-3.63	-2.40	TTHERM_00316330	Protein phosphatase 2C
**Glycosyl hydrolase family**				
g27273	-5.41	-4.85	TTHERM_00283470	Glycosphingolipid Hydrolyzing Enzymes
g22955	-4.30	-4.32	TTHERM_01181990	Glycoside hydrolase, family 27
g20322	-3.94	-1.84	TTHERM_00083400	Glycosyl Hydrolase 25 Enzymes
g12815	-3.86	-2.53	TTHERM_00321640	Glycoside hydrolase, family 35
g18778	-3.85	-2.86	TTHERM_00043770	GAL5 alpha-galactosidase
g15186	-3.76	-5.20	TTHERM_00129820	Beta-hexosaminidase
g11734	-3.63	-4.18	TTHERM_00283480	Glycosphingolipid Hydrolyzing Enzymes
**Kazal domain**				
g21878	-5.15	-3.38	TTHERM_00652530	kazal-type proteinase inhibitor 1
g2538	-4.08	-3.10	TTHERM_00823510	Kazal domain
**Ankyrin repeats**				
g13086	-5.14	-4.00	TTHERM_00655480	Ankyrin repeat
g5144	-4.83	-0.61	TTHERM_00622970	Ankyrin repeat
**EF-hand domain pair**				
g21553	-4.29	-3.91	TTHERM_00037700	EF-hand domain pair
g8146	-3.67	-0.79	TTHERM_00436230	EF-hand domain pair
**Ubiquitin family**				
g9070	-3.97	0.73	TTHERM_00411760	E3 ubiquitin-protein ligase RNF170
g27101	-3.63	-3.76	TTHERM_00113170	Ubiquitin-like domain

g5299	-3.62	-1.40	TTHERM_00578990	ubiquitin-conjugating enzyme family protein
**other protein**				
g6314	-5.69	-0.82	TTHERM_00586840	RAD3L5 type III restriction enzyme res subunit
g11678	-5.52	-5.92	TTHERM_00284040	Coenzyme A pyrophosphatase
g23445	-5.32	-3.36	TTHERM_00833750	THD17 predicted protein
g13191	-5.28	-5.24	TTHERM_00219440	Bacterial Peptidoglycan Processing Enzymes
g23350	-5.12	-3.18	TTHERM_00227860	Patatin-like phospholipase domain
g1938	-5.05	-5.99	TTHERM_00998910	Immunoglobulin-like fold
g4135	-4.99	1.47	TTHERM_00688790	Protein/nucleic acid deglycase
g12405	-4.77	-3.57	TTHERM_00392720	Band-7 stomatin-like
g12139	-4.74	-1.41	TTHERM_00274560	Stress and Tellurium Resistance Protein
g8259	-4.66	-2.45	TTHERM_00455500	JMJ2 JmjC domain protein
g21027	-4.61	-0.83	TTHERM_01100540	Multi antimicrobial extrusion protein
g2236	-4.56	-2.06	TTHERM_00849510	Class I myosin tail homology domain
g18311	-4.37	-4.78	TTHERM_00058590	FBP1 fructose-1-6-bisphosphatase cytosolic protein
g17721	-4.37	-2.87	TTHERM_00211510	GST8 glutathione S-transferase amine-terminal domain protein
g1055	-4.35	-5.20	TTHERM_01109980	Serine/Threonine Dehydratase
g68	-4.34	-8.03	TTHERM_01194730	Choloylglycine hydrolase/NAAA C-terminal
g19398	-4.27	-1.86	TTHERM_00113060	CHS5 chitin synthase
g19144	-4.06	-3.31	TTHERM_00052340	Flavin-containing Monooxygenases
g12223	-3.96	-2.17	TTHERM_00266720	DRAM/TMEM150 Autophagy Modulator
g18412	-3.91	-2.07	TTHERM_00161850	triose-phosphate transporter family protein
g4054	-3.87	-0.41	TTHERM_00852920	TTLL10C tubulin-tyrosine ligase family protein
g139	-3.85	-1.70	TTHERM_00685970	RRM44 polypyrimidine tract-binding protein
g10272	-3.84	-1.27	TTHERM_00329980	Quinoprotein alcohol dehydrogenase-like superfamily

g7857	-3.75	-2.43	TTHERM_00218829	Alpha-aminoadipic semialdehyde synthase
g24531	-3.75	-9.59	TTHERM_01018540	GRL9 granule lattice protein
g4599	-3.72	-2.04	TTHERM_00727830	Adenosine/adenine deaminase
g8557	-3.70	-4.54	TTHERM_00481340	Tyrosyl-DNA phosphodiesterase 2-like

Table S7. The most significantly altered proteins of ST-8 compared to control were arranged on functional groups. ($p < 0.05$)

Functional group	Protein name	GeneName	FOLD CHANGE
**Lipid metabolism**	START-2 domain protein	TTHERM_01084120	42.29
	START domain protein	TTHERM_00569140	1.28
	Cyclopropane-fatty-acyl-phospholipid synthase	TTHERM_00448810	1.28
**RNA-processing**	PRP38 family protein	TTHERM_000836611	7.98
	G-patch domain protein	TTHERM_00046410	2.79
	YbaK/prolyl-tRNA synthetase associated domain	TTHERM_00304350	1.79
	Anticodon-binding domain protein	TTHERM_000922941	1.75
	16S rRNA methyltransferase	TTHERM_00117580	1.46
	ribonuclease Z	TTHERM_01125320	1.45
	RRM domain-containing protein	TTHERM_00046950	1.43
**cGAS-STING**	Type I phosphodiesterase/nucleotide pyrophosphatase	TTHERM_00320270	5.86
**Enables oligosaccharide binding activity**	Chitinase domain-containing protein 1	TTHERM_00729150	3.87
**Transmembrane - transport proteins**	Transmembrane protein	TTHERM_00694400	3.81
	Calcium-translocating P-type ATPase	TTHERM_00522460	3.77
	Protein YIPF	TTHERM_00193890	1.70
	Transmembrane protein	TTHERM_00727740	1.59
	Transporter	TTHERM_00678310	1.58
	Transmembrane protein	TTHERM_00046990	1.46
	Transmembrane protein	TTHERM_00444280	1.44
	Transmembrane protein	TTHERM_00686260	1.44
	Transmembrane amino acid transporter protein	TTHERM_00537220	1.44
	Transmembrane protein	TTHERM_01195950	1.41
	Transmembrane protein	TTHERM_00476900	1.40
	Transmembrane protein	TTHERM_00471150	1.39
	Protein YIPF	TTHERM_000046129	1.38
	Transmembrane protein	TTHERM_00825320	1.38
	ABC1 family protein	TTHERM_01197150	1.34
	Transmembrane protein	TTHERM_00723210	1.32
	Transmembrane protein	TTHERM_00532320	1.30
	MFS transporter	TTHERM_00388160	1.28
	Transmembrane protein	TTHERM_00134960	1.28
	Transmembrane protein	TTHERM_00011030	1.24
	Transmembrane protein	TTHERM_00992690	1.22
	Transmembrane protein	TTHERM_00419920	0.81
	Transmembrane protein	TTHERM_00317270	0.77
	Transmembrane protein	TTHERM_00664000	0.72
	Transmembrane protein	TTHERM_00441700	0.65
**Transmembrane -transport proteins Vesicle formation - membrane trafficking**	PX-SNX-like domain protein	TTHERM_00127270	2.89
	E1-E2 ATPase family protein	TTHERM_00923150	2.09
	Inhibitor of apoptosis-promoting Bax1 protein	TTHERM_000160869	1.38
	Sodium/hydrogen exchanger family protein	TTHERM_00999040	1.37

	Calcium-transporting ATPase	TTHERM_000535709	1.22
**Protein –modification -processing**	Kinase	TTHERM_00758790	2.49
	Serine/Threonine kinase domain protein	TTHERM_00074310	1.74
	Serine/Threonine kinase domain protein	TTHERM_01126380	1.46
	Serine/Threonine kinase domain protein	TTHERM_00624920	1.33
	Serine/threonine-protein phosphatase	TTHERM_00238830	1.21
	Serine/threonine-protein phosphatase	TTHERM_00695560	1.21
	Serine/threonine-protein phosphatase	TTHERM_00431300	0.77
	Palmitoyltransferase	TTHERM_00045030	1.77
	LITAF-like zinc ribbon domain protein	TTHERM_01248930	1.74
	Kinase domain protein	TTHERM_00729010	1.60
	Class V aminotransferase	TTHERM_00418550	1.57
	N-acetylglucosaminylphosphatidylinositol	TTHERM_00471420	1.51
	Kinase domain protein	TTHERM_00384790	1.43
	Kinase domain protein	TTHERM_00344170	1.41
	Kinase domain protein (Fragment)	TTHERM_01670170	1.41
	Kinase domain protein	TTHERM_000537169	1.35
	Protein kinase	TTHERM_00136280	1.41
	Protein kinase	TTHERM_000070849	0.79
	Protein kinase	TTHERM_00600150	0.68
	Alpha/beta superfamily hydrolase	TTHERM_00849190	1.40
	Casein kinase II subunit beta	TTHERM_00780530	1.39
	YEATS domain protein	TTHERM_00561450	1.31
	phosphatidylinositol N-	TTHERM_00449060	1.29
	phosphoinositide 5-phosphatase	TTHERM_00621470	1.25
	GNAT family acetyltransferase	TTHERM_000242129	1.21
	GDSL-like lipase/acylhydrolase	TTHERM_00563920	0.83
	GRAM domain protein	TTHERM_00255670	0.77
	Rhodanese-like domain protein	TTHERM_00486800	0.76
	Armadillo-type fold	TTHERM_00671960	0.60
	Monomeric sarcosine oxidase	TTHERM_00414420	0.20
**Reading proteins**	YTH domain-containing protein	TTHERM_00399440	2.23
**post-translational modifications**	Arginine N-methyltransferase	TTHERM_00219490	2.19
**Oxidative stress**	Cytochrome b5-like heme/steroid	TTHERM_00338510	2.17
	Glutaredoxin-like protein	TTHERM_000312259	1.54
	FAD-binding domain protein	TTHERM_00616550	1.52
	Peroxisomal biogenesis factor 11	TTHERM_00051990	1.49
	Cytochrome P450 monooxygenase	TTHERM_00537280	0.81
	Oxidoreductase, short chain	TTHERM_00462940	1.36
	Oxidoreductase, short chain	TTHERM_00024170	1.27
	Oxidoreductase (Short-chain dehydrogenase	TTHERM_001015921	1.24
	Peroxisomal membrane anchor motif protein	TTHERM_00537280	0.81
**Cell signaling**	Synaptobrevin	TTHERM_00046910	1.73
	Syntaxin-73 protein	TTHERM_00158230	1.71
	FHA domain protein	TTHERM_00194520	1.34
	Dopey, amine-terminal domain protein	TTHERM_00069330	1.30
	C2 domain protein	TTHERM_000471639	1.27

	Saposin-like type B, region 1 protein	TTHERM_00579020	0.65
	Replication stress response regulator SDE2	TTHERM_00216150	0.58
**Motor protein**	Dynactin subunit 5	TTHERM_00637740	1.63
	Dynactin subunit 4	TTHERM_00977540	1.47
**Ribosomes - Translation**	Ribosomal protein S3	rps3	1.61
	Ribosomal protein	rps14	1.41
	Ribosomal subunit protein	TTHERM_00194710	1.26
**Ribosomes – Translation machinery**	RPAP3 monad-binding domain protein	TTHERM_00283980	1.21
**RNA-related - RNA processing**	B-box zinc finger protein	TTHERM_00161790	1.57
**UbiquitinProteasome system**	Ubiquitin fusion degradation protein	TTHERM_00355130	1.52
	ubiquitin-conjugating enzyme	TTHERM_00347960	1.46
	Ubiquitin-fold modifier-conjugating enzyme 1	TTHERM_01108500	1.28
**Vesicle formation - membrane trafficking - Vacuole**	ARF GTPase activator	TTHERM_00837870	1.51
	Rab-GTPase-TBC domain protein	TTHERM_00666440	1.29
	Rab-family small GTPase RabX27	RABX27	1.46
	Rab-family small GTPase RabX21	RABX21	1.43
	Rab-family small GTPase Rab11B	RAB11B	0.81
	Opioid growth factor receptor	TTHERM_00071120	1.44
	Protein YIF1	TTHERM_00195900	1.24
	Trafficking protein particle complex subunit 11	TTHERM_00848130	0.78
	V-type proton ATPase subunit F	TTHERM_00047070	0.73
**Nucleocytoplasmic transport**	Exportin-7 protein	TTHERM_00565580	1.51
	Importin subunit alpha	TTHERM_00079000	1.37
	Importin subunit alpha	TTHERM_00048830	1.31
	SAC3/GANP family protein	TTHERM_00049290	1.26
	Spindle pole body component	TTHERM_00426160	1.24
**Transcription**	General transcription factor IIH subunit	TTHERM_00152050	1.48
	CCR4-NOT transcription complex subunit 11	TTHERM_00473350	1.22
**chimeric proteins**	Leucine rich repeat protein	TTHERM_00284050	1.46
**Proteolysis**	Papain family cysteine protease	TTHERM_00079970	1.42
	Eukaryotic aspartyl protease	TTHERM_00191210	1.41
	Peptidase family M1 protein	TTHERM_00579080	1.20
	NUDIX hydrolase	TTHERM_000050609	0.81
**Cellular cycle**	HCaRG protein	TTHERM_000310868	1.41
	PAPA-like motif protein	TTHERM_00703840	1.37
**DNA-related – Nucleus - Metabolism bases**	Poly [ADP-ribose] polymerase	TTHERM_00006070	1.37
	DNA polymerase delta catalytic subunit	TTHERM_00444660	1.27
	DNA-directed RNA polymerase III subunit RPC3	TTHERM_00989510	1.27
	GTP-binding nuclear protein Ran	P41915	1.23
	Bromodomain protein	TTHERM_00142380	1.20
	Myb-like DNA-binding domain protein	TTHERM_00649290	0.22
**Multifunction**	Tetratricopeptide repeat protein	TTHERM_00327340	1.33
**RNA metabolism -processing**	DEAD-box helicase	TTHERM_00579150	1.33
	RNA recognition motif protein	TTHERM_01015930	1.29
**Energy metabolism Glycolysis - Gluconeogenesis - Cycle ATC Krebs**	Probable cytosolic iron-sulfur protein assembly	TTHERM_00194680	1.32

**mitochondrial biogenesis**	Tim17/tim22/tim23/pmp24 family protein	TTHERM_00289270	1.32
	Ymf77	ymf77	1.31
	rRNA methyltransferase 1, mitochondrial	TTHERM_00840150	1.26
	COX assembly mitochondrial protein	TTHERM_00348340	0.83
**Cell morphology – Cytoskeletal organization**	WD domain, G-beta repeat protein	TTHERM_00030480	1.31
	WD domain, G-beta repeat protein	TTHERM_001006569	0.71
	Katanin p80 WD40 repeat-containing subunit	TTHERM_00497660	0.80
**Antigenic immobilization - Immune system**	S-antigen protein	TTHERM_00497790	1.28
**Uncharacterized protein**	Uncharacterized protein	TTHERM_00502510	7.27
	Uncharacterized protein	TTHERM_00147550	7.08
	Uncharacterized protein	TTHERM_00078980	2.10
	Uncharacterized protein	TTHERM_00301930	1.42
	Uncharacterized protein	TTHERM_01108550	1.41
	Uncharacterized protein	TTHERM_00035290	1.36
	Uncharacterized protein	TTHERM_00780710	1.22
	Uncharacterized protein	TTHERM_00704020	0.78
	Uncharacterized protein	TTHERM_00633140	0.77
	Uncharacterized protein	TTHERM_00685990	0.70

References

1. Hao H, Cao L, Jiang C *et al.* Farnesoid x receptor regulation of the nlrp3 inflammasome underlies cholestasis-associated sepsis. *Cell Metab* 2017;**25** https://doi.org/10.1016/j.cmet.2017.03.007
2. Li D, Li L, Li W *et al.* Elevated o2 alleviated anaerobic metabolism in postharvest winter jujube fruit by regulating pyruvic acid and energy metabolism. *Postharvest Biology and Technology* 2023;**203**:112397. https://doi.org/https://doi.org/10.1016/j.postharvbio.2023.112397
3. Ahmed ME, Hammam ARA, Ali AE-F *et al.* Measurement of carbohydrates and organic acids in varieties of cheese using high-performance liquid chromatography. *Food Sci Nutr* 2023;**11**:2081-85. https://doi.org/10.1002/fsn3.2438
4. Zhang Y, Li Y, Feng Q *et al.* Polydatin attenuates cadmium-induced oxidative stress via stimulating sod activity and regulating mitochondrial function in musca domestica larvae. *Chemosphere* 2020;**248**:126009. https://doi.org/10.1016/j.chemosphere.2020.126009
5. Kraunsøe R, Boushel R, Hansen CN *et al.* Mitochondrial respiration in subcutaneous and visceral adipose tissue from patients with morbid obesity. *J Physiol* 2010;**588**:2023-32. https://doi.org/10.1113/jphysiol.2009.184754
6. Zhang Y, Yuan F, Li P *et al.* Resveratrol inhibits hela cell proliferation by regulating mitochondrial function. *Ecotoxicol Environ Saf* 2022;**241**:113788. https://doi.org/10.1016/j.ecoenv.2022.113788

Table S1. The nucleotide sequences of the primers used in this study. Table S2. qRT-PCR standard-curve parameters. Table S3. Growth kinetic parameters of WT and ST (ST-4, ST-8, ST-12) strains during exponential phase. Table S4. Sequencing and assembly statistics of the transcriptome data from different T. thermophila strains. Table S5. The first 500 genes up-regulated in the ST-8 strain. Table S6. The first 500 genes down-regulated in the ST-8 strain. Table S7. The most significantly altered proteins of ST-8 compared to control were arranged on functional groups.
